# Integrating 72-h BUN/Cr trajectories with interpretable machine learning for 28-day mortality after acute stroke: a dual-center landmark study

**DOI:** 10.3389/fneur.2026.1865384

**Published:** 2026-10-06

**Authors:** Zihai Cheng, Zhongsheng Lu, Shuren Wang, Menghui He, Yiwei Lv, Zhuoma Duoji, Xiaoqing Jin, Wanpeng Fan, Pei Han, Qiang Zhang

**Affiliations:** 1 College of Clinical Medicine Qinghai University, Qinghai University, Xining, China; 2 Department of Neurosurgery, Qinghai Provincial People’s Hospital, Xining, China; 3 Qinghai Provincial People’s Hospital, Xining, China

**Keywords:** 72-h landmark, acute stroke, blood urea nitrogen-to-creatinine ratio, external validation, latent class trajectory model, machine learning

## Abstract

**Background:**

The blood urea nitrogen-to-creatinine ratio (BUN/Cr) may reflect renal perfusion and systemic stress, but its early course after acute stroke is unclear. We identified 72-h BUN/Cr trajectories and assessed their role in interpretable machine-learning evaluation of 28-day mortality.

**Methods:**

This dual-center landmark study included 2,805 ICU patients with acute ischemic stroke, nontraumatic intracerebral hemorrhage, or nontraumatic subarachnoid hemorrhage from the Medical Information Mart for Intensive Care IV (MIMIC-IV) version 3.1. Before modeling, patients were split into a training set (*n* = 1,964) and an internal test set (*n* = 841). The trajectory and prediction models developed in the training set were applied without modification to the internal test set and the Qinghai external validation cohort (*n* = 387). Eight algorithms and prespecified nested models were evaluated using Cox regression, discrimination, calibration, paired comparisons, decision curves, and TreeSHAP.

**Results:**

A quadratic three-class model identified low/stable, high U-shaped, and rising-to-plateau patterns, with mortality rates of 21.6, 35.9, and 39.7%. Adjusted hazard ratios versus Class 1 were 1.87 (95% CI 1.25–2.79) for Class 2 and 1.73 (1.31–2.29) for Class 3. The training-selected Light Gradient Boosting Machine (LightGBM) achieved an AUROC of 0.734 (95% CI 0.695–0.773), a calibration slope of 0.894, and an O/E ratio of 1.015 in the internal test set. In the Qinghai external validation cohort, the AUROC was 0.624 (95% CI 0.559–0.689), the calibration slope was 0.459, and the O/E ratio was 1.920. Adding trajectory class to a model already containing routine clinical and static renal variables changed the AUROC by 0.003 in the internal test set (*p* = 0.515) and −0.014 in the Qinghai external validation cohort (*p* = 0.130), without a clear gain in discrimination.

**Conclusion:**

BUN/Cr trajectories were associated with post-landmark mortality and summarized the three-day renal course. Training-selected LightGBM showed moderate internal discrimination and underwent frozen external evaluation. Trajectory class added modest information beyond routine clinical and static renal variables, while the combined approach provides an interpretable framework for 72-h reassessment.

## Introduction

Stroke remains a leading cause of death and long-term disability. In this study, the acute-stroke population comprised ICU-admitted adults with acute ischemic stroke or nontraumatic hemorrhagic stroke, including intracerebral hemorrhage (ICH) and subarachnoid hemorrhage (SAH). These patients frequently experience impaired consciousness, hemodynamic instability, metabolic disturbance, infection, and multiorgan dysfunction; short-term outcome therefore reflects both neurological injury and the systemic response to acute brain damage (1, 2). Autonomic activation, inflammation, endothelial dysfunction, and altered perfusion can affect the kidney and other organs, while acute kidney injury after stroke is itself associated with adverse outcomes (3–7). Routinely available markers that describe these early changes may therefore add useful information to risk reassessment during the ICU course.

BUN and creatinine reflect related but distinct physiological processes. Creatinine is influenced by glomerular filtration, muscle mass, generation rate, and dilution, whereas BUN is additionally affected by renal perfusion, tubular reabsorption, neurohormonal activation, protein catabolism, and volume status (8–10). BUN/Cr may therefore capture aspects of renal and systemic stress that are not fully represented by either measurement alone. Previous stroke studies linked a higher admission or first-day BUN/Cr value to short-term mortality or unfavorable functional outcome (11, 12). A single time point, however, cannot show whether the ratio remains stable, fluctuates, or continues to change over the next 72 h. We therefore hypothesized that repeated BUN/Cr measurements during the first three ICU days would define prognostically relevant courses, and we combined these courses with interpretable machine learning for structured assessment at a 72-h landmark (13–18).

The study addressed three questions. First, could a parsimonious BUN/Cr trajectory model be derived in the training cohort and applied without refitting to patients in the internal test set and the Qinghai external validation cohort? Second, were the resulting patterns associated with mortality after the 72-h landmark, and did that association remain evident across alternative trajectory specifications and stroke-subtype analyses? Third, did trajectory class add incremental predictive information beyond routine clinical and static renal measurements? This sequence separated phenotype identification, prognostic association, and prediction so that each contribution of the trajectory could be evaluated directly.

## Materials and methods

### Study design and data sources

We performed a retrospective dual-center cohort study with a prespecified 72-h landmark. Development data were obtained from MIMIC-IV version 3.1, a de-identified electronic health record resource from Beth Israel Deaconess Medical Center (19), and the external cohort was assembled at Qinghai Provincial People’s Hospital. Adults admitted to the ICU with acute ischemic stroke, nontraumatic intracerebral hemorrhage (ICH), or nontraumatic subarachnoid hemorrhage (SAH) were identified from diagnosis records. The prespecified diagnosis code set comprised ICD-10-CM I63* and ICD-9-CM 433×1/434×1 for ischemic stroke, ICD-10-CM I61* and ICD-9-CM 431 for ICH, and ICD-10-CM I60* and ICD-9-CM 430 for SAH. The exact no-decimal codes used in MIMIC-IV are listed in Supplementary Table S14. For patients with more than one eligible ICU admission, the first eligible admission was retained.

### Cohort construction and 72-h landmark

MIMIC-IV contained 7,417 adult records with acute ischemic or hemorrhagic stroke, of which 6,992 remained after diagnostic and age screening. Two records lacked ICU outtime and could not be classified at the 72-h landmark; 3,321 patients left the ICU before 72 h and 3,669 remained observable. The landmark cohort additionally required valid paired BUN and creatinine measurements in three non-overlapping windows: T1, 0–24 h; T2, 24–48 h; and T3, 48–72 h. Measurements were harmonized to mg/dL before BUN/Cr calculation. The final strict three-window landmark cohort comprised 2,805 patients. Accordingly, the study evaluates subsequent mortality among patients who remained in the ICU and had the required longitudinal measurements at 72 h (20).

The Qinghai source cohort included 402 ICU patients with acute ischemic or hemorrhagic stroke. After application of the corresponding eligibility and three-window renal-measurement requirements, 387 patients were available for fixed trajectory assignment and external evaluation. The primary outcome in both cohorts was the locked indicator of death by day 28 after ICU admission, finalized before application of any prediction model.

To characterize whether the 72-h observation and measurement-completeness requirements altered the composition of the analytic cohort, we performed a *post hoc* descriptive selection analysis. The strict MIMIC-IV landmark cohort was used as the reference and compared with three mutually exclusive groups from the screened cohort: patients still observable at 72 h but lacking complete paired T1-T3 measurements, patients who left the ICU before 72 h with an ICU-death flag, and patients who left before 72 h without that flag. We summarized baseline characteristics, illness-severity measures, and recorded day-28 mortality, and calculated absolute standardized mean differences relative to the landmark cohort (Supplementary Table S16). This analysis was intended to describe the direction and magnitude of selection associated with cohort construction; absence of an ICU-death flag was not interpreted as evidence of clinical stability.

### Patient-level split and development sequence

Before trajectory modeling, feature selection, or preprocessing, the MIMIC-IV landmark cohort was split once at the patient level in a 70:30 ratio, jointly stratified by mortality and stroke subtype. The training set contained 1,964 patients with 460 events, and the internal test set contained 841 patients with 197 events. Trajectory fitting and candidate selection, preprocessing estimates, LASSO selection, hyperparameter optimization, algorithm selection, and threshold selection were conducted using training data. The fixed models were then evaluated once in the internal test set and subsequently applied to the external cohort. The training-only development sequence, prevalidation locking, and frozen evaluation are summarized in Figure 1.

**Figure 1:**
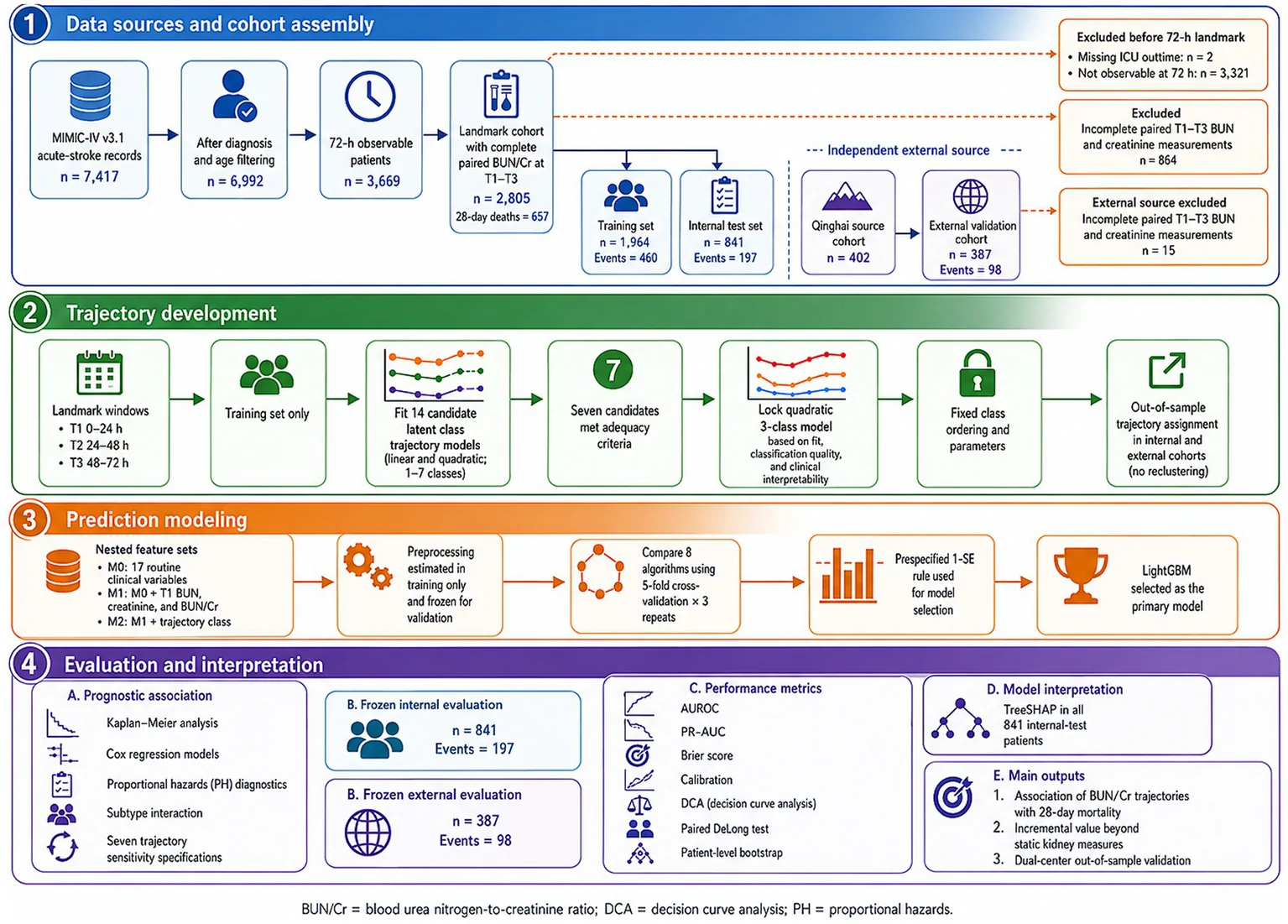
Study design and analytic framework. Trajectory and prediction models were developed in the training cohort and locked before the internal test was opened. Internal-test and Qinghai patients received only out-of-sample assignment from the fixed trajectory model and frozen prediction-model evaluation, without reclustering, model reselection, or recalibration.

### Training-only latent class trajectory modeling

Latent class mixed models were fitted to T1-T3 BUN/Cr in the training cohort using the lcmm framework (21, 22). Linear and quadratic time functions were combined with one through seven latent classes to form 14 prespecified candidates. Multi-class models used repeated random initializations to reduce dependence on local optima. Mortality was not used in model selection. Candidates were assessed for convergence, BIC, entropy, minimum class size, class-specific average posterior probability, odds of correct classification, agreement between assigned and model-estimated class proportions, and the proportion of patients with maximum posterior probability at least 0.70. The primary model was chosen by jointly considering fit, class separation, classification quality, subgroup size, parsimony, and clinical interpretability. All candidates meeting the prespecified adequacy criteria were retained for sensitivity analysis.

### Out-of-sample trajectory-class assignment

The quadratic three-class model was fitted in the full training cohort, after which the number of classes, trajectory parameters, and class order were fixed. For each internal-test or Qinghai patient, the T1-T3 BUN/Cr measurements were used to calculate posterior membership probabilities for the three classes, and the class with the highest probability was assigned. Classification quality was evaluated separately in the training, internal-test, and external cohorts. Neither validation cohort contributed to trajectory refitting, class-number selection, or trajectory-shape updating.

### Predictor architecture, LASSO, and missing data

The prediction architecture was nested to distinguish routine clinical information, static renal measurements, and trajectory information. M0 contained 17 routine clinical predictors (full list in Supplementary Table S1) and did not include T1 BUN, T1 creatinine, or T1 BUN/Cr. M1 added these three static renal measurements, and M2 additionally included the locked three-class trajectory variable. LASSO feature selection was conducted within the training workflow, with the penalty chosen by the one-standard-error rule (23, 24), after which the final predictor architecture was fixed for model development.

Machine-learning analyses used tidymodels workflows. Median imputation for numeric variables, handling of missing and novel categorical levels, and dummy encoding or normalization when required by an algorithm were estimated within each analysis fold of the training resamples. After development, the final preprocessing workflow was fitted in the full training cohort and applied without modification to the internal test set and the Qinghai external validation cohort. Pre-imputation missing counts and percentages for all analysis variables in the MIMIC-IV landmark cohort and the Qinghai external validation cohort are reported in Supplementary Table S15. Most variables had less than 5% missingness; delirium was missing in 451 of 2,805 MIMIC-IV patients (16.1%). Two alternative handling strategies were prespecified before internal-test access: fold-specific mode imputation or omission of delirium from M1 and M2. Both analyses retained the selected LightGBM hyperparameters and assessed whether the main conclusions depended on how delirium missingness was handled (Supplementary Table S13).

### Association analyses and diagnostics

The association between the locked trajectory class and mortality was evaluated in the combined MIMIC-IV landmark cohort after outcome-independent trajectory fitting and assignment. Follow-up began at the 72-h landmark. The primary Cox proportional-hazards model adjusted for trajectory class, age, sex, stroke subtype, initial Glasgow Coma Scale, T1 BUN, T1 creatinine, and T1 BUN/Cr. Class 1 was the reference, and results are reported as hazard ratios (HRs) with 95% confidence intervals.

Proportional-hazards assumptions were evaluated with scaled Schoenfeld residuals (25), multicollinearity with VIF/GVIF, and the linear form of continuous covariates with prespecified functional-form analyses. The primary adjusted association was first estimated in the pooled MIMIC-IV landmark cohort. We then tested the overall trajectory-by-stroke-subtype interaction and reported Kaplan–Meier curves and Cox models separately for ischemic and hemorrhagic stroke. Robustness of the trajectory association was further examined across seven class-number and time-form specifications that met the prespecified adequacy criteria (Supplementary Tables S5–S8).

### Machine-learning development and algorithm selection

Eight algorithms were prespecified: naive Bayes, decision tree, random forest, support vector machine, k-nearest neighbors, neural network, XGBoost, and LightGBM. All algorithms were evaluated with the same patient-level five-fold cross-validation repeated three times in the training set. Preprocessing and hyperparameter evaluation were nested within resampling. A prespecified one-standard-error hierarchy based on training cross-validated AUROC selected the algorithm before the internal test set was accessed. The selected LightGBM model was then fitted with fixed hyperparameters for M0, M1, and M2, and patient-level out-of-fold predictions supplied the training-derived classification thresholds (26).

### Clinical score comparators

SAPS II and SOFA were included as prespecified secondary clinical comparators (27–29). The primary incremental prediction question was whether M2 improved performance relative to M1.

### Performance measures and statistical comparisons

Performance was assessed across four complementary domains. Discrimination was summarized by AUROC with 95% confidence intervals and PR-AUC; overall probability error by the Brier score; and calibration by plots, intercept, slope, mean predicted risk, observed event rate, and the observed-to-expected ratio (30, 31). Decision-curve analysis compared standardized net benefit across threshold probabilities (32). Sensitivity, specificity, positive predictive value, negative predictive value, accuracy, and F1 were additionally reported at the frozen training-derived threshold.

Because models were evaluated in the same patients, AUROC differences were tested with paired DeLong methods (33). Patient-level paired bootstrap resampling provided 95% confidence intervals for differences in AUROC and Brier score. Prespecified nested comparisons were M1 versus M0, M2 versus M1, and M2 versus M0. Unless otherwise stated, tests were two-sided and *p* < 0.05 was considered statistically significant.

### Model interpretation

Native LightGBM TreeSHA*p* values were calculated for all 841 internal-test patients (34). The main analysis included a beeswarm summary, mean absolute SHAP ranking, and representative waterfall plots. Supplementary dependence plots described the fitted relationships between leading continuous predictors and SHAP contributions, and force plots provided complementary patient-level visualizations (Supplementary Figures S5, S6).

## Results

### Cohort flow and cross-center characteristics

Among 7,417 MIMIC-IV acute-stroke records, 6,992 remained after diagnostic and age screening; two lacked ICU outtime and could not be classified at the 72-h landmark. A total of 3,321 patients left the ICU before 72 h, including 387 with a documented ICU-death flag, and 3,669 remained observable. Requiring paired BUN and creatinine measurements in all three time windows yielded a final landmark cohort of 2,805 patients, including 657 day-28 deaths (23.4%). The fixed split produced 1,964 training patients with 460 events and 841 internal-test patients with 197 events. In Qinghai, 402 patients were screened, 387 met the three-window requirements, and 98 (25.3%) died by day 28 (Figure 2).

**Figure 2:**
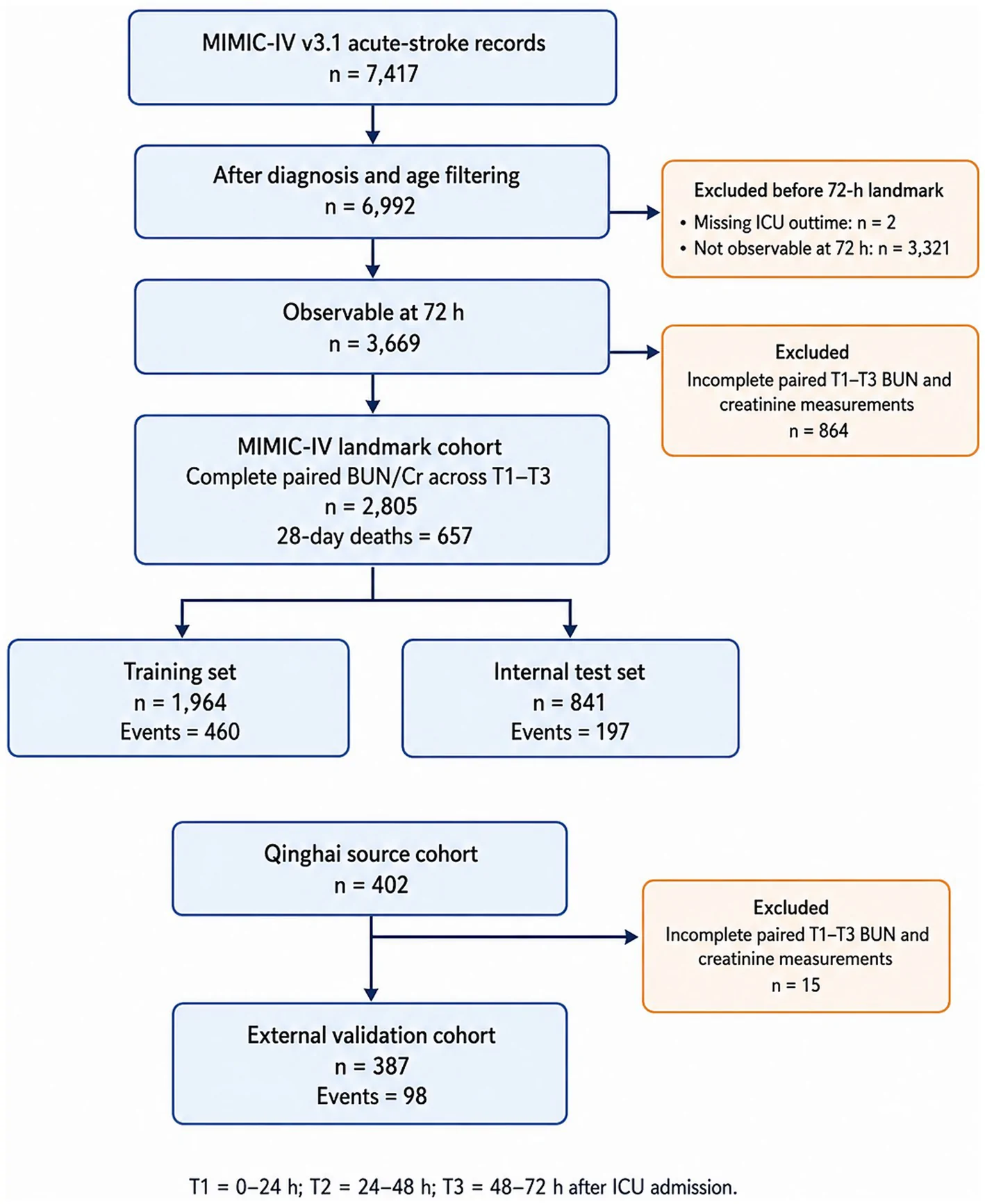
Cohort construction. Flow diagram of MIMIC-IV v3.1 acute ischemic or hemorrhagic stroke records, formation of the 72-h landmark cohort, the training/internal-test split, and construction of the Qinghai external-validation cohort. T1, 0–24 h; T2, 24–48 h; T3, 48–72 h after ICU admission.

To characterize selection at the 72-h landmark, the strict landmark cohort was compared with the three excluded groups (Supplementary Table S16). Patients who remained observable but had incomplete T1-T3 measurements had a nearly identical recorded day-28 mortality proportion to the landmark cohort (203/864 [23.5%] vs. 657/2,805 [23.4%]) and were similar on many baseline measures; the largest reported differences were in delirium documentation (SMD 0.289) and SOFA score (SMD 0.195). Patients who left before 72 h with an ICU-death flag were older, more often had hemorrhagic stroke, and had higher SAPS II and SOFA values than the landmark cohort (SMDs 0.380, 0.471, 0.701, and 0.344, respectively); all 387 had recorded death by day 28. Patients who left before 72 h without an ICU-death flag had lower recorded day-28 mortality (536/2,934 [18.3%]), lower SAPS II and SOFA values, and larger differences in acute kidney injury and delirium documentation (SMDs 0.318, 0.509, 0.939, and 0.940, respectively). Absence of an ICU-death flag was not interpreted as evidence of clinical stability.

Overall event proportions were similar in the MIMIC-IV landmark and Qinghai cohorts, but case mix differed substantially. Qinghai patients were younger (median 57.0 vs. 67.7 years), more often male (71.3% vs. 54.0%), and more often had hemorrhagic stroke (82.4% vs. 43.1%). Median initial GCS was lower externally (8 vs. 15; absolute SMD 1.854), whereas median SOFA was higher (7 vs. 4; SMD 0.938). Red blood cell count was higher (4.7 vs. 4.0 ×10^12/L; SMD 0.978), anion gap was lower (10.0 vs. 14.0 mmol/L; SMD 0.870), and first-24-h urine output was higher (2,078 vs. 1,575 mL; SMD 0.230). These findings show that neurological status, stroke composition, organ dysfunction, and laboratory profiles differed substantially between centers.

Additional diagnostic and recording variables also differed, including delirium documentation (40.2% versus 63.6%); such differences may reflect both case mix and center-specific recording practices. As context for overall illness severity in the MIMIC-IV landmark cohort, 77.75% of patients had SOFA scores of 0–6 and only 2.53% had scores of 13 or higher (Table 1 and Figure 3). Together with differences in age, initial neurological status, stroke-subtype composition, organ dysfunction, and laboratory distributions, these findings characterize the Qinghai cohort as a cross-regional evaluation population with a substantially different case mix.

**Table 1:** Baseline, model-related, trajectory, and outcome characteristics of the MIMIC-IV landmark and Qinghai external cohorts.

Characteristic	MIMIC-IV landmark cohort (*n* = 2,805)	Qinghai external cohort (*n* = 387)	Absolute SMD
Age, years	67.7 [56.0, 78.5]	57.0 [52.0, 66.0]	0.583
Initial GCS	15.0 [14.0, 15.0]	8.0 [6.0, 11.0]	1.854
Heart rate, beats/min	82.0 [71.0, 95.0]	80.0 [67.5, 96.0]	0.046
Respiratory rate, breaths/min	18.0 [16.0, 22.0]	20.0 [18.0, 21.0]	0.255
Anion gap, mmol/L	14.0 [12.0, 16.0]	10.0 [7.9, 12.3]	0.870
Total calcium, mg/dL	8.6 [8.1, 9.0]	8.4 [8.0, 8.8]	0.178
Potassium, mmol/L	4.0 [3.6, 4.4]	3.8 [3.5, 4.1]	0.425
Glucose, mg/dL	132.0 [109.0, 166.0]	126.8 [108.1, 153.2]	0.102
White blood cell count, 10^9/L	11.1 [8.4, 14.5]	10.9 [8.5, 13.1]	0.149
Red blood cell count, 10^12/L	4.0 [3.4, 4.4]	4.7 [4.2, 5.4]	0.978
Urine output in first 24 h, mL	1575.0 [989.5, 2369.2]	2077.5 [1302.5, 2772.5]	0.230
T1 BUN, mg/dL	17.0 [12.0, 24.0]	15.5 [12.2, 19.4]	0.212
T1 creatinine, mg/dL	0.90 [0.70, 1.20]	0.85 [0.71, 1.06]	0.021
T1 BUN/Cr ratio	17.62 [13.75, 22.73]	17.25 [13.70, 22.27]	0.044
SAPS II-based score	34.0 [26.0, 43.0]	37.0 [30.0, 46.5]	0.196
SOFA-based score	4.0 [2.0, 6.0]	7.0 [5.0, 9.0]	0.938
Male sex	1,516 (54.0%)	276 (71.3%)	0.363
Hemorrhagic stroke	1,208 (43.1%)	319 (82.4%)	0.891
Congestive heart failure	619 (22.1%)	9 (2.3%)	0.633
Chronic renal disease	490 (17.5%)	27 (7.0%)	0.325
Severe liver disease	74 (2.6%)	44 (11.4%)	0.347
Delirium	1,497 (63.6%); missing 451	155 (40.2%); missing 1	0.612
Acute kidney injury	2,467 (88.0%)	334 (86.3%)	0.049
CRRT	151 (5.4%)	15 (3.9%)	0.072
Trajectory class 1	2,499 (89.1%)	305 (78.8%)	0.283
Trajectory class 2	92 (3.3%)	24 (6.2%)	0.283
Trajectory class 3	214 (7.6%)	58 (15.0%)	0.283
Recorded outcome event	657 (23.4%)	98 (25.3%)	0.044

Continuous variables are shown as median [IQR] and categorical variables as *n* (%). SMD denotes absolute standardized mean difference. Where data were missing, percentages use the observed denominator.

**Figure 3:**
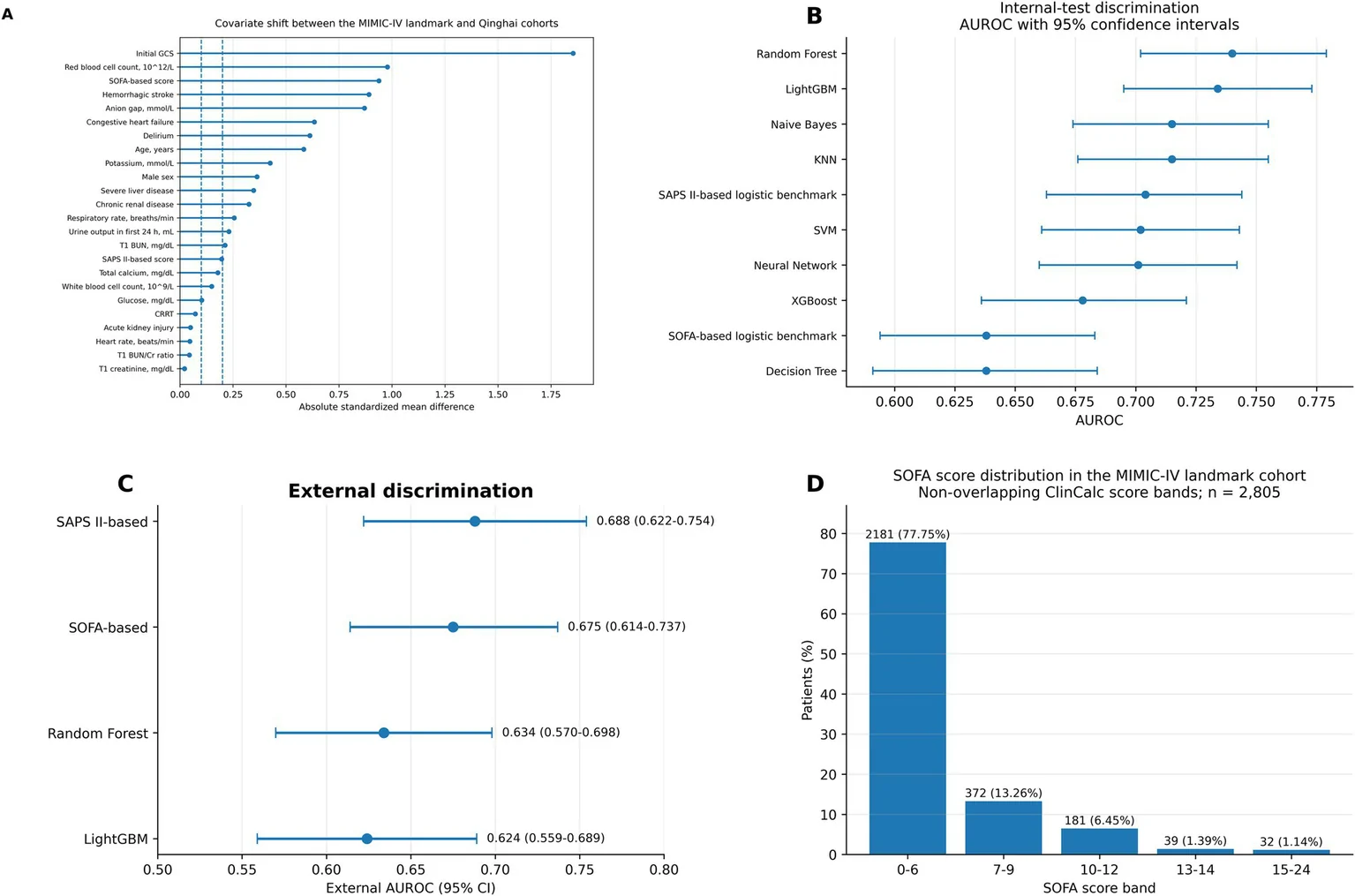
Cross-center transportability and performance context. **(A)** Absolute standardized mean differences between the MIMIC-IV landmark and Qinghai cohorts. Dashed lines indicate conventional 0.10 and 0.20 reference values. **(B)** Internal-test AUROCs and 95% confidence intervals. **(C)** Selected external AUROCs and 95% confidence intervals. **(D)** Distribution of SOFA-based score bands in the MIMIC-IV landmark cohort.

### Trajectory-model selection and classification quality

Seven candidate trajectory models met the prespecified adequacy criteria: three linear models with two to four classes and four quadratic models with two to five classes. At each shared class count, the quadratic model had a lower BIC and higher entropy than the corresponding linear model, supporting the quadratic time form. Within the quadratic family, BIC decreased from 37,471.4 for two classes to 37,195.5 for three, 36,994.2 for four, and 36,908.3 for five. Classification quality was strongest for the three-class solution: entropy was 0.868, 95.2% of patients had a maximum posterior probability of at least 0.70, the smallest class represented 3.26% of training patients, and the lowest class-specific average posterior probability was 0.852. The four- and five-class solutions further reduced BIC but had lower entropy (0.679 and 0.710), lower high-confidence assignment (77.3% and 75.2%), and smaller minimum classes (2.70% and 2.44%) (Table 2; Figure 4; Supplementary Table S2).

**Table 2:** Outcome-independent selection metrics for eligible quadratic LCTM candidates.

Quadratic classes	BIC	Entropy	Smallest class, %	Minimum class APP	Max posterior ≥0.70, %	Role
2	37,471.4	0.765	8.35	0.892	95.98	Sensitivity
3	37,195.5	0.868	3.26	0.852	95.21	Primary
4	36,994.2	0.679	2.70	0.786	77.34	Sensitivity
5	36,908.3	0.710	2.44	0.776	75.20	Sensitivity

**Figure 4:**
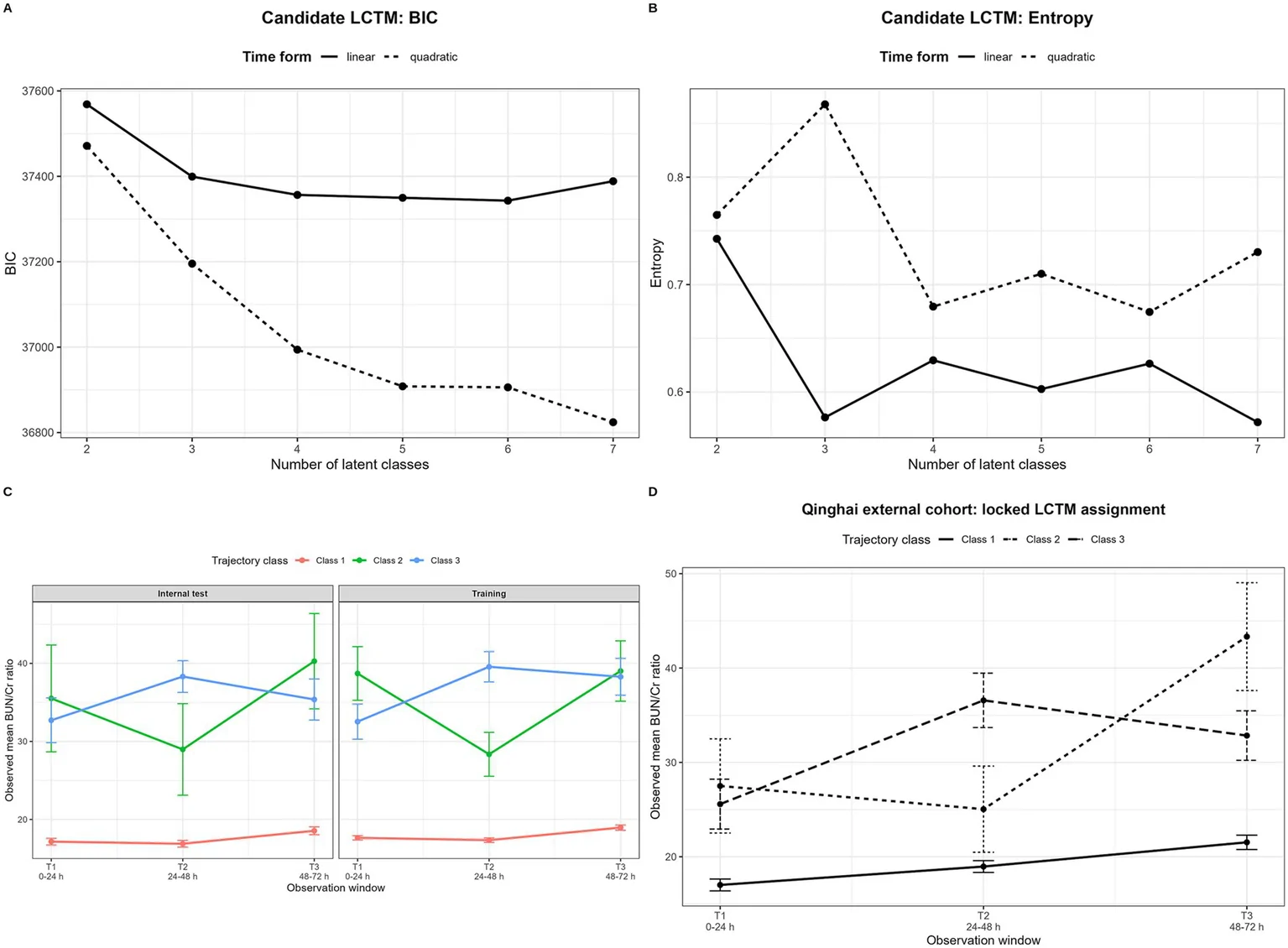
Training-only LCTM selection and out-of-sample trajectory assignment. **(A)** Bayesian information criterion across candidate class numbers and time forms. **(B)** Entropy across candidates. **(C)** Observed mean BUN/Cr trajectories in the training and internal-test cohorts under the locked quadratic three-class model. **(D)** Observed trajectories after fixed-parameter assignment in the Qinghai cohort. The broad low, U-shaped, and rising/elevated ordering was retained externally, although exact magnitudes and late-window behavior differed by center.

The quadratic three-class model was therefore selected without using mortality. The two-class model retained high classification certainty but combined the U-shaped and persistently elevated courses into one broader group. The four- and five-class models improved statistical fit at the cost of smaller and less clearly separated classes. The three-class solution preserved high assignment certainty while distinguishing low/stable, high U-shaped, and rising-to-plateau courses. The three eligible linear models and the remaining eligible quadratic models were all retained for sensitivity analyses, so the main association did not depend on treating the selected structure as the only plausible representation.

### Trajectory phenotypes and out-of-sample reproducibility

In the combined MIMIC-IV landmark cohort, Class 1 included 2,499 patients (89.1%) and remained low with a modest T3 increase (mean BUN/Cr 17.5, 17.2, and 18.8 at T1-T3). Class 2 included 92 patients (3.3%) and followed a high U-shaped course (37.7, 28.5, and 39.4). Class 3 included 214 patients (7.6%) and rose from an already elevated level before remaining high (32.6, 39.2, and 37.4). Training and internal-test means showed the same ordering and closely corresponding shapes, while class-specific assigned posterior probabilities remained high in both datasets (Figure 4; Supplementary Tables S3, S4).

The external cohort also preserved the broad ordering of the fixed phenotypes, although the observed curves were not numerically identical. Class 1 remained the lowest trajectory and rose gradually (17.0, 19.0, and 21.5). Class 2 retained the U-shaped configuration, with an early decline followed by a marked T3 rebound (27.5, 25.0, and 43.3). Class 3 rose sharply at T2 and remained elevated at T3 (25.6, 36.6, and 32.8). Fixed assignment placed 305, 24, and 58 Qinghai patients in Classes 1–3, with mean assigned posterior probabilities of 0.938, 0.893, and 0.867. These results support reproducible out-of-sample recognition of the three general phenotypes while also showing center-specific differences in class prevalence and exact trajectory magnitude.

### Trajectory classes and mortality

Mortality increased from 21.6% in Class 1 (539/2,499) to 35.9% in Class 2 (33/92) and 39.7% in Class 3 (85/214). Kaplan–Meier curves separated after the landmark and remained ordered through follow-up (log-rank *p* < 0.0001; Figure 5). In the prespecified adjusted Cox model, Class 2 had an HR of 1.87 (95% CI 1.25–2.79; *p* = 0.002) and Class 3 an HR of 1.73 (1.31–2.29; *p* < 0.001) relative to Class 1 (Table 3). The two adverse patterns therefore conveyed similar excess risk despite different temporal shapes: one showed renewed elevation after an interim decline, whereas the other remained persistently elevated after an early rise.

**Figure 5:**
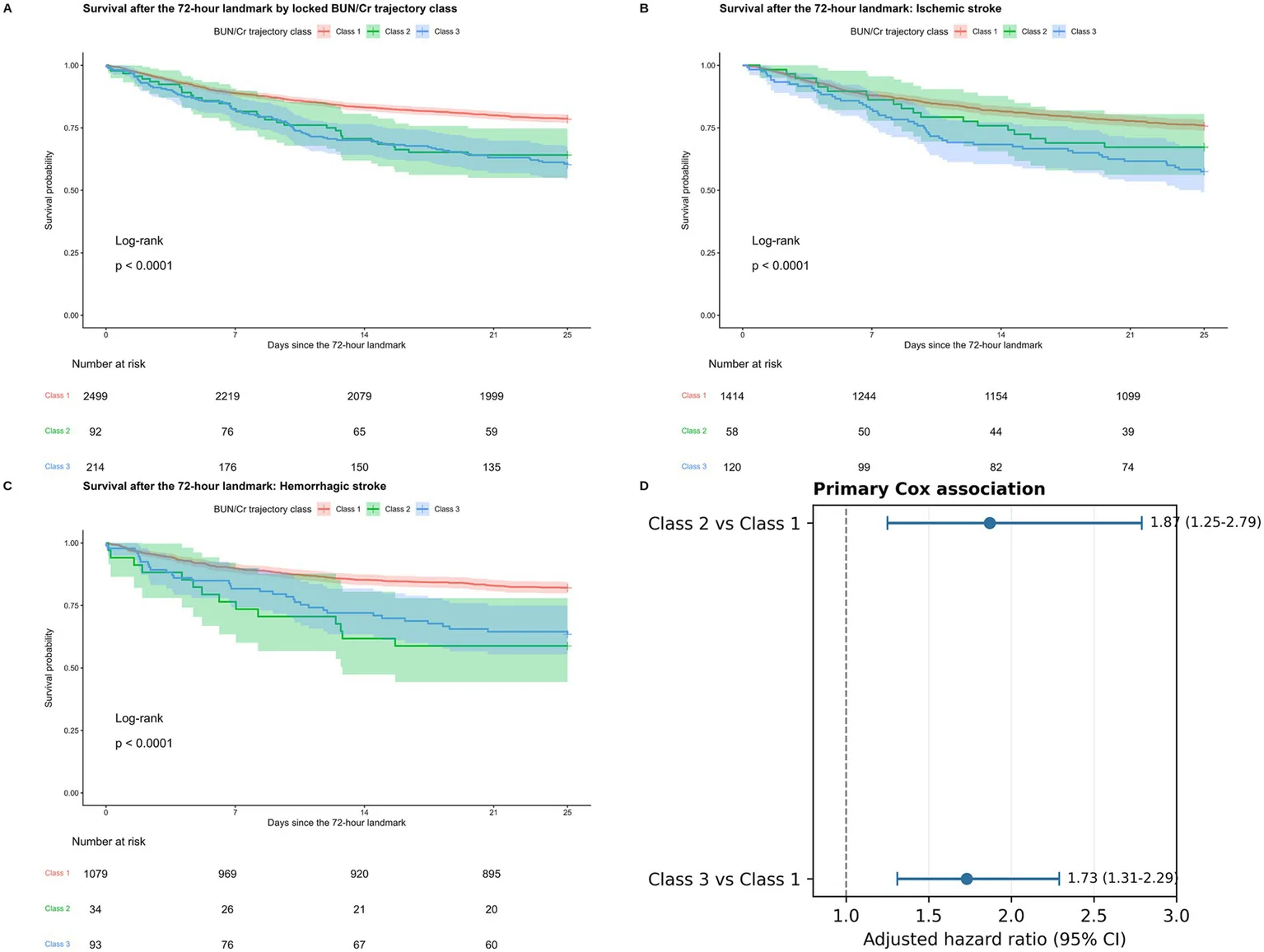
Prognostic association of BUN/Cr trajectory classes after the 72-h landmark. Kaplan–Meier curves and risk tables for **(A)** the full MIMIC-IV landmark cohort, **(B)** ischemic stroke, and **(C)** hemorrhagic stroke. **(D)** Adjusted hazard ratios for Class 2 and Class 3 versus Class 1 from the prespecified primary Cox model.

**Table 3:** Distribution, event rates, and adjusted Cox associations of the three BUN/Cr trajectory classes.

Trajectory class	Clinical description	*n* (%)	Events, *n* (%)	Mean BUN/Cr T1/T2/T3	Adjusted HR vs class 1 (95% CI)	*p*-value
Class 1	Low, stable with modest late rise	2,499 (89.1)	539 (21.6)	17.5/17.2/18.8	Reference	—
Class 2	High U-shaped	92 (3.3)	33 (35.9)	37.7/28.5/39.4	1.87 (1.25–2.79)	0.002
Class 3	Higher, rising to plateau	214 (7.6)	85 (39.7)	32.6/39.2/37.4	1.73 (1.31–2.29)	<0.001

Proportional-hazards tests assessed whether relative hazards could be treated as approximately constant during follow-up. Neither the global test (*p* = 0.133) nor the trajectory-class test (*p* = 0.494) indicated material time variation, supporting interpretation of the Class 2 and Class 3 HRs as average post-landmark relative hazards. The stroke-subtype term showed time variation (*p* = 0.0017), indicating that the relative risk associated with ischemic versus hemorrhagic stroke was not constant over follow-up. We therefore also reported subtype-stratified Kaplan–Meier curves and subtype-specific Cox models. This finding concerns the time pattern of the subtype main effect; whether trajectory effects differed by subtype was evaluated separately with the trajectory-by-subtype interaction. The maximum VIF/GVIF-derived value was 1.66, with no evidence of problematic multicollinearity (Supplementary Tables S5, S6).

### Robustness across trajectory specifications and stroke subtypes

The trajectory-by-stroke-subtype interaction was tested first to determine whether the association differed between ischemic and hemorrhagic stroke. The interaction *p*-value was 0.383, providing no evidence of clear effect modification. The pooled Cox model was therefore retained as the primary inference, and subtype-specific analyses were used to describe how the association appeared within each stroke type.

Within ischemic stroke, the joint test of the Class 2 and Class 3 coefficients was *p* = 0.106. This global test evaluates whether mortality risk differs across the three trajectory classes within ischemic stroke; it is not a proportional-hazards or time-dependence test. Class 3 versus Class 1 had an HR of 1.49 (95% CI 1.03–2.15; *p* = 0.034), whereas Class 2 had an HR of 1.34 (0.79–2.27; *p* = 0.271) and its confidence interval crossed 1.

Within hemorrhagic stroke, the global trajectory-class test was *p* < 0.001. HRs for Class 2 and Class 3 versus Class 1 were 3.03 (95% CI 1.62–5.66; *p* < 0.001) and 2.22 (1.46–3.39; *p* < 0.001), respectively. Although the point estimates were larger in hemorrhagic stroke, within-subtype HRs and *p*-values cannot replace the formal interaction test. Because the trajectory-by-stroke-subtype interaction was *p* = 0.383, these results do not establish a stronger trajectory effect in hemorrhagic than in ischemic stroke.

All seven eligible trajectory specifications were used to assess whether the pooled association depended on a particular class number or time form. They comprised three linear models with two to four classes and four quadratic models with two to five classes. The global trajectory-class test was significant in every specification, with *p*-values from 9.8 × 10^−8^ to 0.00463.

Because the number and clinical meaning of classes were not equivalent across specifications, sensitivity was evaluated with each model’s global trajectory-class test rather than by forcing class-specific HRs to align. The pooled trajectory-mortality association was observed across multiple linear and quadratic representations, showing that the principal conclusion did not depend on the quadratic three-class model alone (Supplementary Table S8).

### Training-only algorithm selection

With the candidate predictors determined via LASSO screening and nested architecture construction (Figure 6), we then proceeded to select the optimal algorithm using cross-validation. In five-fold cross-validation repeated three times, LightGBM achieved the highest mean AUROC, 0.740184 (SE 0.005123). The prespecified one-standard-error candidate rule defined the lower bound as the best mean AUROC minus the standard error of that best estimate, yielding 0.735061. This rule incorporated resampling uncertainty and avoided treating a very small mean difference as a stable advantage. LightGBM was the only algorithm above the bound; random forest ranked second at 0.734534 (SE 0.005309), 0.000527 below the threshold.

**Figure 6:**
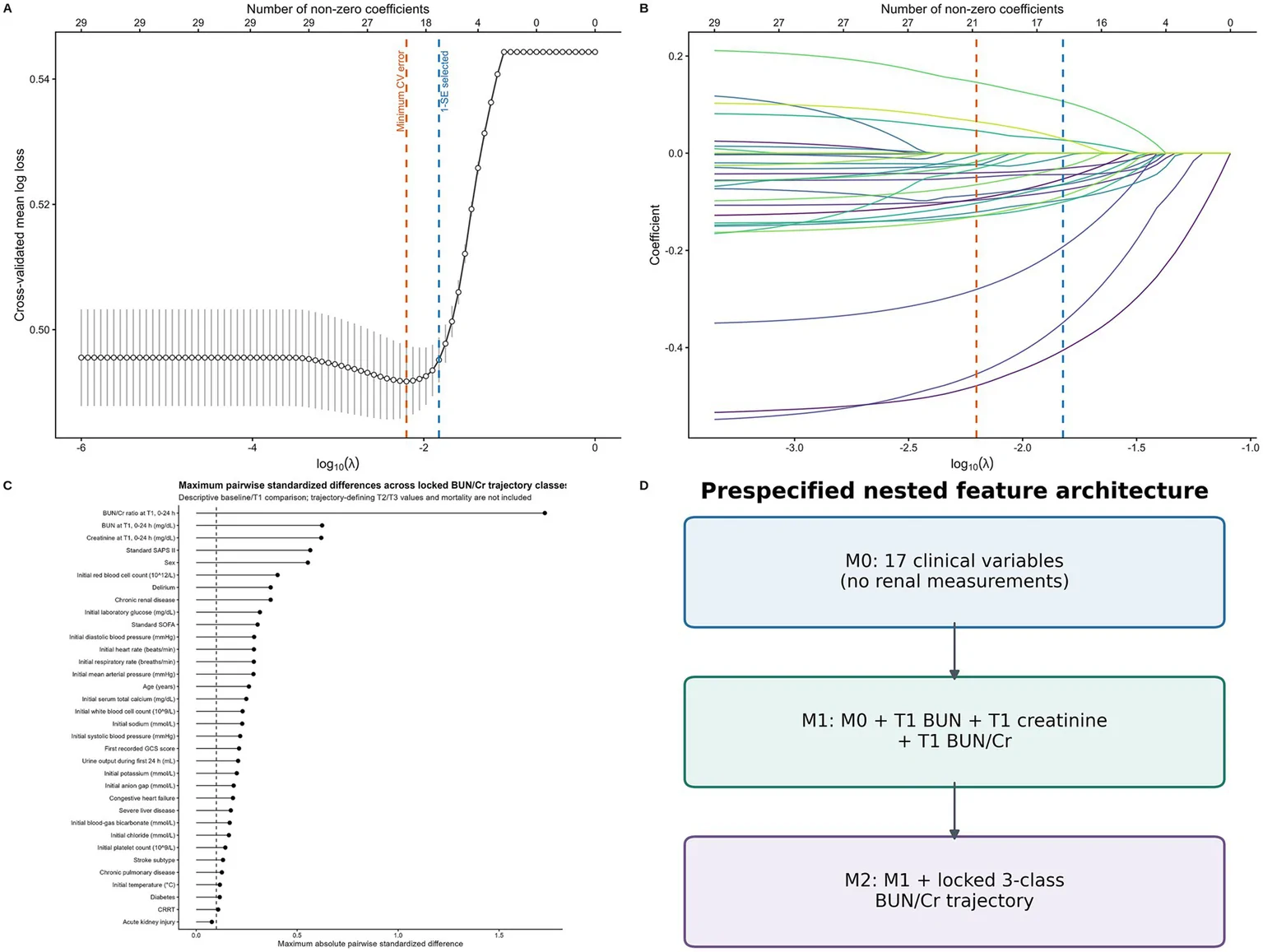
Feature selection and nested prediction architecture. **(A)** Cross-validation curve for the fold-internal LASSO penalty. **(B)** Coefficient paths. **(C)** Between-class standardized differences used to characterize the trajectory phenotypes. **(D)** Nested feature architecture: M0 includes 17 prespecified clinical variables, M1 adds T1 BUN, creatinine, and BUN/Cr, and M2 adds the locked trajectory class.

The out-of-fold Brier scores of the two tree-ensemble models were nearly identical (LightGBM 0.158249; random forest 0.158064). The calibration intercept/slope was 0.030/0.900 for LightGBM and −0.040/1.170 for random forest. Because the one-standard-error candidate set contained only LightGBM, no subsequent tie-breaking comparison was needed. LightGBM was fixed as the primary model before internal-test access; the internal and external cohorts were subsequently used only for evaluation, not for algorithm selection.

### Internal validation and clinical-score comparisons

In the one-time internal test, LightGBM M2 achieved an AUROC of 0.734 (95% CI 0.695–0.773), PR-AUC of 0.455, and Brier score of 0.157. The calibration intercept was 0.022, indicating close agreement between average predicted and observed risk. The slope was 0.894, close to but slightly below 1, suggesting mildly over-dispersed predicted risks with only a small departure from ideal calibration. At the locked training threshold, sensitivity was 0.695, specificity 0.648, PPV 0.376, and NPV 0.874 (Table 4; Figures 7, 8). Overall, the model showed moderate discrimination, accurate average risk estimation, and positive net benefit over part of the displayed threshold range.

**Table 4:** Internal and external performance of nested LightGBM models and score-based benchmarks.

Cohort/model	AUROC (95% CI)	PR-AUC	Brier	Calibration intercept	Calibration slope	O/E
Internal LightGBM M0	0.730 (0.692–0.769)	0.448	0.158	0.016	1.052	1.011
Internal LightGBM M1	0.731 (0.692–0.770)	0.463	0.157	0.013	1.117	1.009
Internal LightGBM M2	0.734 (0.695–0.773)	0.455	0.157	0.022	0.894	1.015
Internal SAPS II-based	0.704 (0.663–0.744)	0.400	0.165	0.033	1.018	1.023
Internal SOFA-based	0.638 (0.594–0.683)	0.348	0.172	0.019	1.081	1.014
External LightGBM M0	0.620 (0.555–0.685)	0.353	0.196	0.804	0.621	1.831
External LightGBM M1	0.638 (0.574–0.702)	0.355	0.194	0.773	0.736	1.794
External LightGBM M2	0.624 (0.559–0.689)	0.341	0.199	0.896	0.459	1.920
External SAPS II-based	0.688 (0.622–0.754)	0.496	0.169	0.019	1.292	1.013
External SOFA-based	0.675 (0.614–0.737)	0.454	0.178	−0.237	2.096	0.845

**Figure 7:**
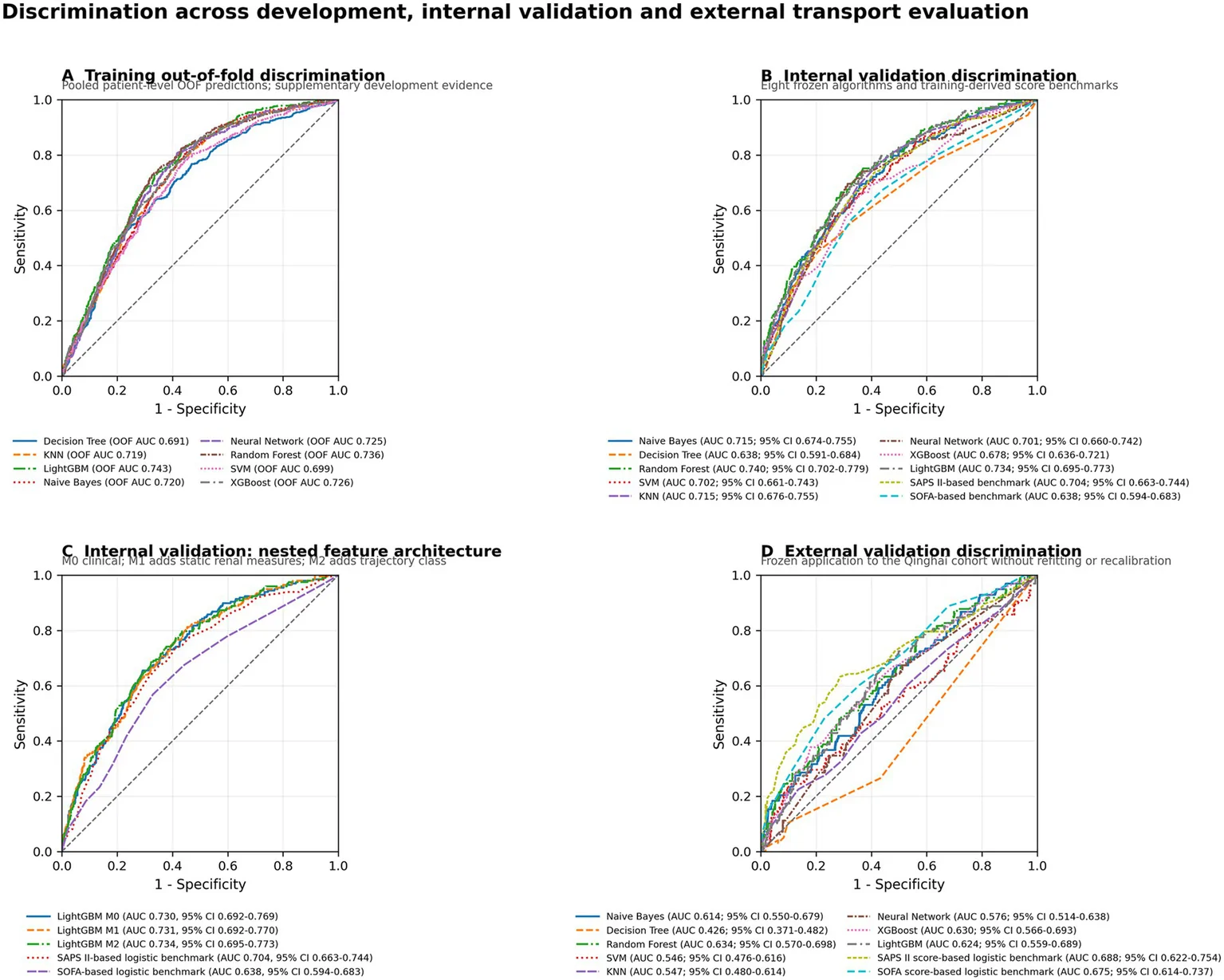
Discrimination across development, internal validation, and external transport evaluation. **(A)** Pooled patient-level training out-of-fold ROC curves for eight algorithms; these curves are development evidence rather than final test performance. **(B)** Internal-test ROC curves for the frozen algorithms and training-derived score benchmarks, with AUROC and 95% confidence intervals in the legend. **(C)** Internal M0/M1/M2 and score-benchmark comparison. **(D)** External ROC curves from frozen application to the Qinghai cohort.

**Figure 8:**
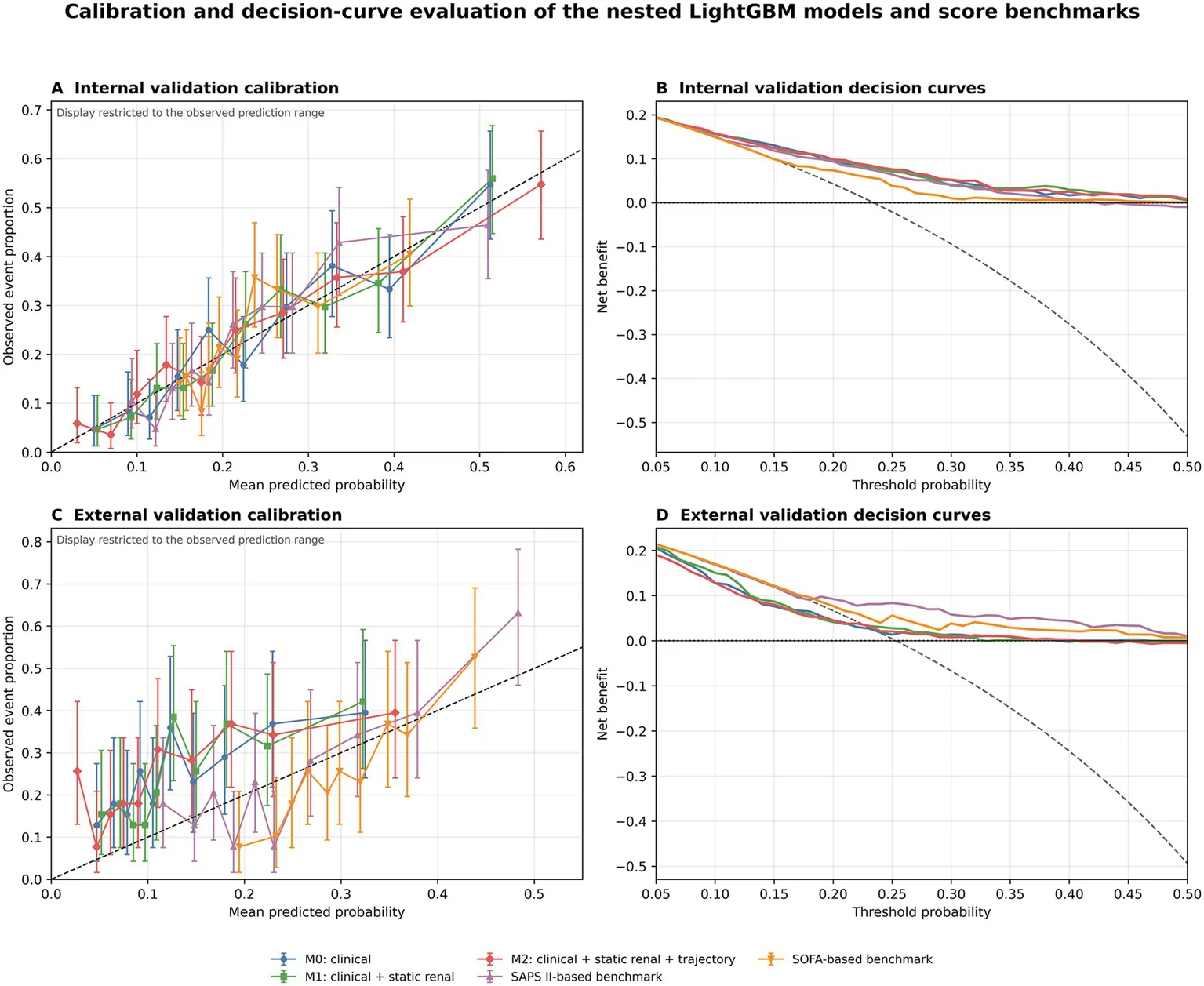
Calibration and decision-curve evaluation. Calibration plots and decision curves for M0, M1, M2, and the score-based benchmarks in the **(A,B)** internal test and **(C,D)** Qinghai external cohort. Calibration panels are displayed over the observed prediction range rather than extending across an empty 0–1 range. Points show grouped observed event proportions with confidence intervals; dashed lines indicate ideal calibration or the treat-all strategy, as applicable.

Random forest achieved an internal AUROC of 0.740 and LightGBM 0.734; the paired difference was −0.006 (*p* = 0.499), with no clear discrimination difference. The fact that the random-forest AUROC in this internal sample was slightly above its repeated-CV training mean is not contradictory: the training value is an average across resamples, whereas the internal AUROC is a single estimate in an independent finite sample, and either can move upward or downward with case mix and sampling variation. The AUROC difference between LightGBM and the SAPS II comparator was 0.031 (*p* = 0.168), also not statistically significant. These comparisons do not show that LightGBM discriminated better than random forest or the SAPS II comparator in the internal test. LightGBM remained the primary model because algorithm selection had already been completed under the prespecified training rule; the internal test evaluated that choice rather than reopening model selection.

### Incremental contribution of renal measurements and trajectory class

Internal AUROCs for M0, M1, and M2 were 0.730, 0.731, and 0.734. The M2-versus-M1 AUROC difference was 0.003 (paired DeLong *p* = 0.515; patient-level bootstrap 95% CI −0.007 to 0.012), and the Brier-score difference was 0.00006 (95% CI −0.00237 to 0.00264). Their ROC, calibration, and decision curves largely overlapped. Thus, after routine clinical variables and T1 BUN, creatinine, and BUN/Cr were already available, adding trajectory class did not materially improve internal risk ranking, probability accuracy, or net benefit. The class label remained an interpretable description of the three-day course, but much of its predictive information was shared with existing variables (Table 5; Figures 7, 8).

**Table 5:** Paired incremental comparisons among M0, M1, and M2.

Cohort/comparison	ΔAUROC	DeLong P	Bootstrap 95% CI for ΔAUROC	ΔBrier	Bootstrap 95% CI for ΔBrier
Internal M1-M0	0.0007	0.902	−0.0110 to 0.0110	−0.00093	−0.00308 to 0.00130
Internal M2-M1 (primary)	0.0031	0.515	−0.0065 to 0.0123	0.00006	−0.00237 to 0.00264
Internal M2-M0	0.0038	0.513	−0.0080 to 0.0149	−0.00087	−0.00392 to 0.00208
External M1-M0	0.0181	0.110	−0.0035 to 0.0399	−0.00180	−0.00474 to 0.00091
External M2-M1 (primary)	−0.0141	0.130	−0.0330 to 0.0032	0.00434	0.00113 to 0.00763
External M2-M0	0.0041	0.748	−0.0203 to 0.0296	0.00254	−0.00167 to 0.00628

### TreeSHAP interpretation

TreeSHA*p* values were calculated for all 841 internal-test patients. Age had the largest mean absolute contribution (0.487), followed by glucose (0.296), delirium status (0.207), total calcium (0.189), first-24-h urine output (0.149), white blood cell count (0.141), and T1 BUN (0.127) (Figure 9; Supplementary Table S12). The beeswarm plot showed that older age, higher glucose, higher white blood cell count, and several abnormal renal and metabolic measurements commonly shifted predictions toward higher mortality risk, although the magnitude of each feature’s contribution varied substantially across patients.

**Figure 9:**
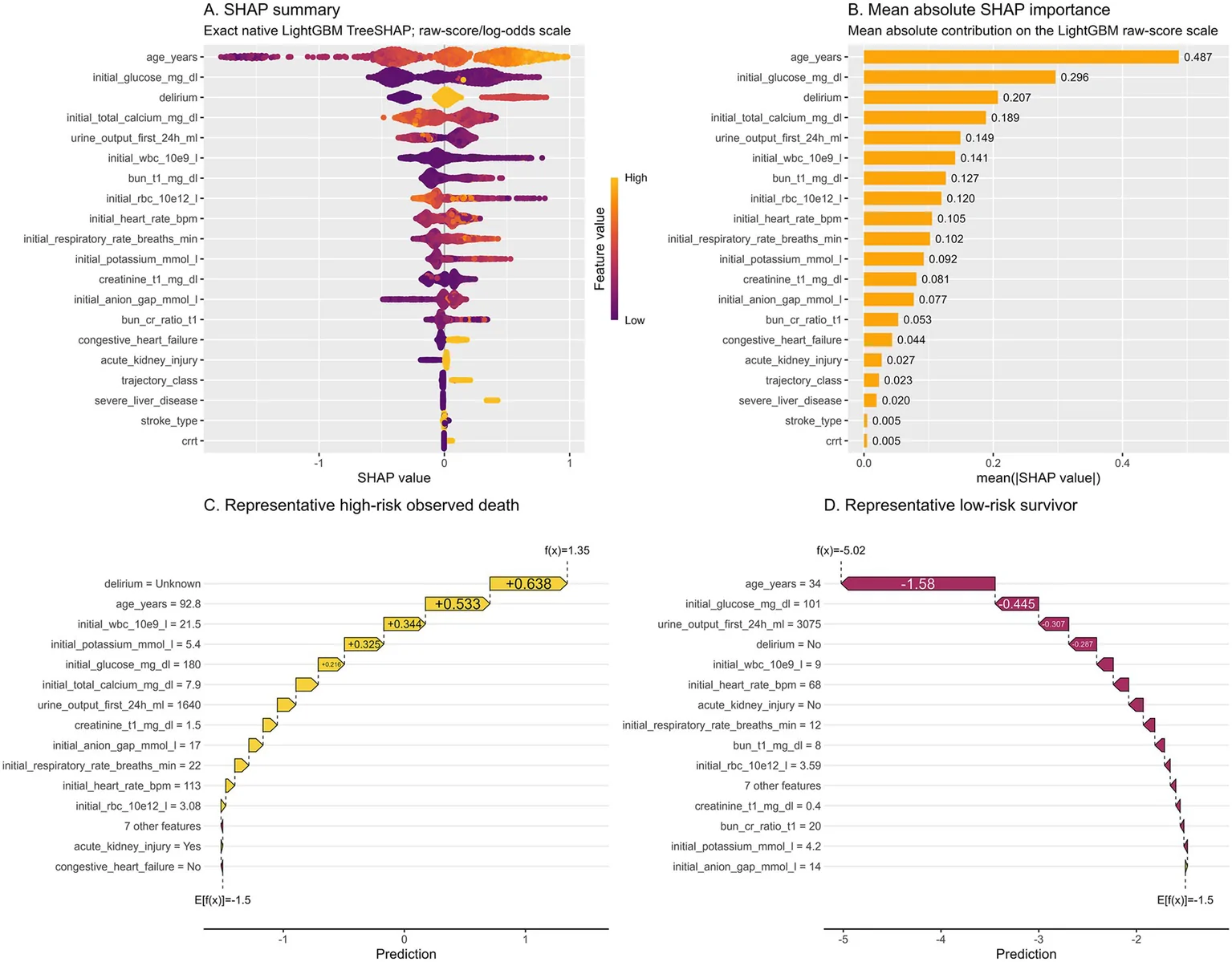
Native LightGBM TreeSHAP interpretation in the internal test set. **(A)** Beeswarm summary of feature contributions on the mortality log-odds scale. **(B)** Mean absolute SHAP importance for the principal original predictors displayed in the figure. **(C,D)** Representative waterfall plots for a higher-risk death case and a lower-risk survival case.

Supplementary dependence plots further described the fitted relationships. The contribution of age generally increased with age. Glucose and white blood cell count did not follow simple straight-line relationships with model output, indicating that their contributions varied across value ranges and in combination with other predictors. Lower total calcium was usually associated with contributions toward higher risk, whereas higher first-24-h urine output generally shifted the model toward lower risk. LightGBM therefore integrated information on physiological reserve, metabolic state, inflammation, renal function, and acute brain dysfunction. Global importance summarizes average contribution across patients; an individual prediction still needs to be interpreted from the complete feature profile.

The waterfall plots showed how individual feature contributions accumulated from the model baseline to each prediction. In the representative higher-risk death case, age 92.8 years, delirium recorded as Unknown, white blood cell count 21.5 × 10^9^/L, potassium 5.4 mmol/L, and glucose 180 mg/dL all increased the mortality log-odds. In the representative lower-risk survivor, age 34 years, glucose 101 mg/dL, first-24-h urine output 3,075 mL, and absence of delirium all decreased the model output. These cases illustrate how the same model produces patient-specific explanations from different combinations of clinical features; global rankings cannot substitute for individual interpretation.

Trajectory class had a lower mean SHAP contribution than the leading clinical and biochemical variables. This finding was consistent with the direct M2-versus-M1 comparison: the trajectory was prognostically associated with mortality at the population level, but after static renal and other clinical information had entered the model, the label contributed relatively little additional patient-level predictive information.

### External validation

All external predictions used the frozen training models and thresholds, and no model parameters were updated in the Qinghai cohort. LightGBM M2 achieved an AUROC of 0.624 (95% CI 0.559–0.689), PR-AUC of 0.341, and Brier score of 0.199 (Table 4; Figure 7). Observed mortality was 0.253 and mean predicted risk was 0.132 (O/E = 1.92); the calibration intercept was 0.896, indicating overall underestimation of risk. The calibration slope was 0.459, indicating that lower- and higher-risk patients were separated less clearly in Qinghai than in the internal test. Decision-curve analysis showed no consistent net-benefit advantage for M2 over M1 across the displayed thresholds (Figure 8).

External AUROCs for M0, M1, and M2 were 0.620, 0.638, and 0.624. The M2-versus-M1 AUROC difference was −0.014 (paired DeLong *p* = 0.130), with a patient-level bootstrap 95% CI of −0.033 to 0.003. Because the interval crossed zero, the external data did not show improved patient ranking after trajectory class was added. The M2 Brier score exceeded M1 by 0.00434 (bootstrap 95% CI 0.00113–0.00763), indicating greater probability error. Together with the decision curves, these results show that trajectory class did not add external predictive benefit beyond the routine clinical and static renal variables in M1.

This result was not caused by failure to assign external patients to trajectory classes. Mean posterior probabilities in Qinghai were 0.938, 0.893, and 0.867, indicating generally good fixed-model assignment. Thus, recognizability and predictive increment were separate findings: the three patterns remained assignable in Qinghai, but the class label did not improve external prediction after M1 had already incorporated T1 BUN, creatinine, BUN/Cr, and other clinical variables. Potential contributions from information overlap, class size, and cross-center case-mix differences are considered in the Discussion.

## Discussion

### Principal findings

The findings can be summarized in four steps. First, a fixed three-class model developed in the training set identified low/stable, high U-shaped, and rising-to-plateau BUN/Cr courses in both internal and external patients. Second, the latter two patterns were associated with higher mortality after the 72-h landmark in the pooled MIMIC-IV cohort, and the global association remained evident across all seven eligible trajectory specifications. Third, training-selected LightGBM showed moderate discrimination and reasonable calibration in the internal test; direct M2-versus-M1 comparisons showed that trajectory class summarized the three-day course but shared substantial patient-level predictive information with static renal and other clinical variables. Finally, the Qinghai evaluation confirmed that the fixed trajectory model could classify new patients while showing that discrimination and calibration of the complete risk model changed under a different case mix.

### Pathophysiological interpretation of the trajectories

BUN/Cr does not correspond to a single kidney mechanism; it reflects the combined behavior of BUN and creatinine under several physiological influences. After acute stroke, autonomic and inflammatory responses, reduced intake, dysphagia, fever, and hemodynamic fluctuation may lower effective circulating volume and renal perfusion. Sympathetic activation, the renin-angiotensin-aldosterone system, and vasopressin signaling can then increase proximal tubular urea reabsorption, while protein catabolism during critical illness increases urea generation. BUN may therefore rise before or more prominently than creatinine (3–6, 10, 35, 36).

Creatinine is additionally influenced by muscle mass, generation rate, and dilution from fluid therapy. Resuscitation, changing circulatory status, and evolving kidney injury can therefore alter BUN and creatinine at different rates (8, 9, 35). The three-day BUN/Cr course consequently reflects a changing balance among perfusion, catabolic demand, neuroendocrine stress, renal reserve, and treatment-related volume shifts. Its value is to describe how early physiology evolves, rather than to identify one isolated causal pathway.

The three classes can be interpreted cautiously within this framework. A low/stable course may represent relatively preserved balance among renal perfusion, catabolic load, and volume status. A high U-shaped course may begin with hypovolemia, neurohormonal activation, or pre-renal stress, improve temporarily after early treatment, and rise again when catabolism, recurrent hemodynamic stress, kidney dysfunction, or complications persist. A rising-to-plateau course suggests sustained or progressive abnormality. Although their shapes differ, both higher-risk patterns may mark reduced renal reserve, fluid and electrolyte disturbance, and worsening multiorgan function. These are physiological interpretations of observational patterns, not direct proof of a specific mechanism.

The SHAP ranking further placed the BUN/Cr trajectory within a broader risk profile. Age was the leading feature and may capture reduced physiological reserve, accumulated comorbidity, and lower tolerance of secondary insults. Glucose had a nonlinear contribution; stress hyperglycemia after stroke is linked to catecholamine and cortisol responses, oxidative stress, endothelial dysfunction, and inflammation (37). Lower urine output can indicate reduced effective circulating volume, impaired renal perfusion, or lower filtration (7, 35), whereas higher white blood cell counts may reflect post-stroke inflammation and myeloid immune activation (5, 36). Together, these variables describe physiological reserve, metabolic response, inflammation, and organ perfusion, complementing the time-varying signal summarized by BUN/Cr trajectory.

Delirium reflects acute brain dysfunction and often accompanies neurological injury, infection, metabolic disturbance, and treatment exposure; systematic evidence links post-stroke delirium to mortality and unfavorable neurological outcomes (38). Total calcium also requires critical-care context because it is influenced by albumin, acid–base status, kidney function, and other organ dysfunction. A previous ICU cohort of patients with intracerebral hemorrhage found that lower total calcium was associated with increased risk of death during hospitalization and during the ICU stay (39). The principal predictors therefore are not isolated signals: together they describe neurological, metabolic, inflammatory, circulatory, and renal status, while BUN/Cr trajectory supplies one time-varying component of that broader profile.

### Relationship between prognostic association and predictive contribution

Population-level prognostic association and patient-level predictive increment address different questions. The primary Cox model asks whether trajectory phenotypes are associated with different subsequent hazards after adjustment for other covariates. The M2-versus-M1 comparison asks whether the class label further improves patient ranking or probability estimates after T1 BUN, creatinine, BUN/Cr, and other clinical variables are already available. Because the label is derived from the same renal measurements and compresses three continuous observations into one category, much of its information overlaps with the static renal variables in M1. Significant adjusted HRs and a small AUROC difference can therefore coexist: the former supports prognostic stratification, whereas the latter indicates limited independent predictive increment beyond variables already in the model.

The incremental contribution of trajectory class is also affected by class size. In MIMIC-IV, Class 1 represented 89.1% of patients, whereas Classes 2 and 3 together represented only 10.9%, leaving fewer observations for learning stable interactions involving the higher-risk classes. In Qinghai, the three broad patterns and high posterior assignment probabilities were retained, but class prevalence, absolute BUN/Cr levels, baseline event risk, and other clinical characteristics differed from MIMIC-IV. These changes do not prevent class assignment, but they may change the mortality risk represented by a class and its relationship with other predictors.

Trajectory identifiability and predictive increment should therefore be evaluated separately. Clear assignment in Qinghai supports the out-of-sample recognizability of the three patterns. The absence of an M2 advantage means that, in this external cohort, the label did not add patient-level predictive information beyond static renal and other clinical variables. These results are compatible and do not establish that static measurements are generally preferable to dynamic trajectories.

### Meaning of the LightGBM selection

Algorithm selection used a prespecified one-standard-error candidate rule. The candidate lower bound was the highest mean cross-validated AUROC minus the standard error of that best estimate. If several algorithms met the bound, out-of-fold Brier score, calibration slope, calibration intercept, and AUROC stability were compared sequentially. This approach incorporated sampling variation in repeated cross-validation and reduced the chance of changing algorithms because of a very small mean AUROC difference. LightGBM achieved a mean AUROC of 0.740184 (SE 0.005123), yielding a candidate lower bound of 0.735061. Random forest achieved 0.734534 (SE 0.005309), 0.000527 below the bound. Their out-of-fold Brier scores were nearly identical (0.158249 and 0.158064); the LightGBM calibration slope was closer to 1 (0.900 vs. 1.170), and both intercepts were close to 0 (0.030 and −0.040). Because LightGBM was the only algorithm above the prespecified bound, it was fixed as the primary model before internal-test access.

In the one-time internal test, random forest achieved an AUROC of 0.740 and LightGBM 0.734 (paired *p* = 0.499), indicating similar discrimination. The study retained the training-selected LightGBM as the primary model and reported random forest and the paired comparison in full. This respected the model-selection rule established before validation while accurately showing that the internal test did not demonstrate a clear difference between the two tree-ensemble methods.

### External validation and clinical implications

The Qinghai cohort differed from MIMIC-IV in age, stroke subtype, initial neurological status, organ-failure scores, and several hematologic and metabolic measurements. Despite these differences, the locked training LCTM assigned the three trajectory classes with high posterior probabilities, showing that the low/stable, high U-shaped, and rising-to-plateau patterns remained identifiable in independent patients. Reliable class assignment, however, does not necessarily mean that mortality-risk relationships learned in the training center will remain unchanged elsewhere. Differences in baseline risk, clinical practice, and relationships among predictors can alter the correspondence between predicted probabilities and observed outcomes. Consistent with this distinction, LightGBM M2 had an external AUROC of 0.624, mean predicted mortality of 13.2% versus observed mortality of 25.3% (O/E = 1.92), and a calibration slope of 0.459. The Qinghai cohort was used to evaluate the model without local parameter updating, so these results describe the performance of the original model in a new setting. The preserved trajectory assignment and the change in risk-model performance highlight complementary aspects of external evaluation and support local performance assessment before clinical use (40, 41).

The intended clinical setting is reassessment at the 72-h landmark rather than admission triage. For patients with acute stroke who remain in the ICU and have three renal measurements, BUN/Cr trajectory organizes the laboratory history into three readily interpretable courses: low/stable, high U-shaped, and rising-to-plateau.

When a patient follows a high U-shaped or rising-to-plateau course, clinicians may review urine output, volume status, renal perfusion, metabolic abnormalities, and evolving organ dysfunction in a structured manner. Trajectory information does not replace neurological examination, imaging, or established ICU scores, but it adds the three-day course that a single laboratory value cannot show. Before use in another center, discrimination, calibration, and potential net benefit should be assessed in local patients (42, 43).

### Strengths

A principal strength was the order of development and validation. Patient splitting preceded LCTM fitting, so internal-test trajectories were assigned entirely out of sample. The final class structure was selected from 14 candidates using outcome-independent criteria, and all seven eligible alternatives entered sensitivity analyses. This design assessed both the classification quality of the chosen phenotype structure and the robustness of its mortality association to alternative model specifications.

A second strength was the nested M0/M1/M2 architecture, which separated routine clinical information, static renal measurements, and trajectory class so that the central incremental question was tested directly rather than inferred from feature importance. Eight algorithms were compared within the same repeated-resampling framework, and the algorithm and threshold were fixed before validation. Performance assessment covered AUROC, PR-AUC, Brier score, calibration, paired DeLong tests, bootstrap differences, and decision curves. Association analyses included proportional-hazards diagnostics, VIF/GVIF, functional-form assessment, alternative trajectory specifications, a formal stroke-subtype interaction, and subtype-specific analyses. The dual-center design additionally evaluated both reproducibility of fixed trajectory assignment and the behavior of the complete risk model under a different case mix.

### Limitations

Several limitations define the scope of interpretation.

First, the 72-h landmark and complete-measurement requirements defined a conditional population. The descriptive comparison showed that the incomplete-measurement group had a similar recorded day-28 mortality proportion, whereas the early-departure groups had different clinical and outcome profiles. This comparison describes the selection introduced by cohort construction but does not remove it. The findings therefore apply to patients who remain observable with complete T1-T3 measurements at 72 h, rather than to admission triage or outcomes before the landmark.

Second, subtype analyses described the pooled association within ischemic and hemorrhagic stroke and were not two independent confirmatory studies. The interaction *p*-value of 0.383 did not detect clear effect modification, but some higher-risk subtype classes contained few patients and events; the study therefore had limited ability to detect small subtype differences, and a non-significant interaction does not prove identical effects.

Third, proportional-hazards diagnostics did not show clear time variation in the trajectory-class effects, so the primary HRs can be interpreted as average relative hazards during follow-up. The stroke-subtype main effect was time-varying and could not be summarized by one constant coefficient across the entire follow-up. Subtype-stratified Kaplan–Meier curves were therefore used to display survival patterns within ischemic and hemorrhagic stroke, and subtype-specific Cox models quantified trajectory HRs separately within each subtype rather than relying on one time-varying subtype coefficient. These analyses remained supportive, and the small sizes of some subtype classes limited precision; they did not replace the formal interaction test.

Fourth, imaging lesion characteristics, detailed treatment intensity, and time-varying therapeutic exposures were unavailable.

Finally, external validation was conducted in one regional center with a different case mix. The Qinghai cohort assessed the model without local parameter updating. Additional multicenter evaluation and, where appropriate, independently assessed local updating would help clarify performance across clinical settings.

## Conclusion

Among ICU patients with acute stroke who remained observable at 72 h, high U-shaped and rising-to-plateau BUN/Cr trajectories were associated with higher 28-day mortality. Older age, higher glucose, delirium, lower total calcium, lower urine output, higher white blood cell count, and higher T1 BUN jointly formed the principal risk information used by the model. LightGBM integrating clinical, biochemical, and trajectory features showed moderate internal discrimination and underwent frozen external evaluation. BUN/Cr trajectory provided an intuitive dynamic renal signal for 72-h reassessment; however, once routine clinical and static renal variables were included, trajectory class offered a modest additional predictive contribution.

## Data Availability

The original contributions presented in the study are included in the article/Supplementary material, further inquiries can be directed to the corresponding authors.
