# Supplementary material for: Integrating 72-h BUN/Cr trajectories with interpretable machine learning for 28-day mortality after acute stroke: a dual-center landmark study
Source: Front Neurol. 2026 Oct 6;17:1865384. doi: 10.3389/fneur.2026.1865384 (PMC13680161; doi:10.3389/fneur.2026.1865384)
Supplement: Supplementary file 1 [file Supplementary_file_1.DOCX]

Supplementary Material

# Supplementary Tables

## Supplementary Table S1. Nested predictor architecture.

| **Feature set** | **Predictor count** | **Information layer** | **Added/contained variables** |
| --- | --- | --- | --- |
| M0 | 17 | Routine clinical predictors | Age; stroke subtype; initial heart rate; respiratory rate; anion gap; total calcium; potassium; glucose; WBC; RBC; first-24-hour urine output; congestive heart failure; chronic renal disease; severe liver disease; delirium; acute kidney injury; CRRT |
| M1 | 20 | M0 + static renal measurements | T1 BUN; T1 creatinine; T1 BUN/Cr |
| M2 | 21 | M1 + dynamic phenotype | Locked three-class 72-hour BUN/Cr trajectory |

## Supplementary Table S2. All 14 training-only LCTM candidates.

| **Time form** | **Classes** | **BIC** | **Entropy** | **Minimum class, %** | **Minimum APP** | **Max PP >=0.70, %** | **Eligible** |
| --- | --- | --- | --- | --- | --- | --- | --- |
| linear | 1 | 38,560.9 | 1.000 | 100.00 | 1.000 | 100.00 | No |
| linear | 2 | 37,568.6 | 0.743 | 9.16 | 0.876 | 95.21 | Yes |
| linear | 3 | 37,399.4 | 0.576 | 4.74 | 0.791 | 77.95 | Yes |
| linear | 4 | 37,356.5 | 0.629 | 4.58 | 0.732 | 73.12 | Yes |
| linear | 5 | 37,349.8 | 0.603 | 2.75 | 0.677 | 62.93 | No |
| linear | 6 | 37,343.0 | 0.626 | 2.55 | 0.683 | 60.69 | No |
| linear | 7 | 37,388.6 | 0.572 | 2.85 | 0.509 | 46.69 | No |
| quadratic | 1 | 38,467.6 | 1.000 | 100.00 | 1.000 | 100.00 | No |
| quadratic | 2 | 37,471.4 | 0.765 | 8.35 | 0.892 | 95.98 | Yes |
| quadratic | 3 | 37,195.5 | 0.868 | 3.26 | 0.852 | 95.21 | Yes |
| quadratic | 4 | 36,994.2 | 0.679 | 2.70 | 0.786 | 77.34 | Yes |
| quadratic | 5 | 36,908.3 | 0.710 | 2.44 | 0.776 | 75.20 | Yes |
| quadratic | 6 | 36,905.9 | 0.674 | 1.07 | 0.733 | 66.75 | No |
| quadratic | 7 | 36,824.2 | 0.730 | 1.73 | 0.711 | 75.25 | No |

## Supplementary Table S3. Class-specific posterior assignment quality.

| **Cohort** | **Class** | **n** | **Proportion, %** | **Mean assigned PP** | **Median assigned PP** | **PP >=0.70, %** |
| --- | --- | --- | --- | --- | --- | --- |
| Training | Class 1 | 1749 | 89.1 | 0.959 | 0.990 | 97.0 |
| Training | Class 2 | 64 | 3.3 | 0.892 | 0.982 | 84.4 |
| Training | Class 3 | 151 | 7.7 | 0.852 | 0.914 | 79.5 |
| Internal test | Class 1 | 750 | 89.2 | 0.960 | 0.991 | 96.0 |
| Internal test | Class 2 | 28 | 3.3 | 0.868 | 0.916 | 82.1 |
| Internal test | Class 3 | 63 | 7.5 | 0.875 | 0.966 | 82.5 |
| Qinghai external | Class 1 | 305 | 78.8 | 0.938 | 0.983 | 96.1 |
| Qinghai external | Class 2 | 24 | 6.2 | 0.893 | 0.986 | 79.2 |
| Qinghai external | Class 3 | 58 | 15.0 | 0.867 | 0.940 | 81.0 |

## Supplementary Table S4. Observed mean BUN/Cr values by cohort and assigned trajectory class.

| **Cohort** | **Class** | **T1** | **T2** | **T3** |
| --- | --- | --- | --- | --- |
| Internal test | Class 1 | 17.2 | 16.9 | 18.5 |
| Internal test | Class 2 | 35.5 | 29.0 | 40.3 |
| Internal test | Class 3 | 32.7 | 38.3 | 35.4 |
| Training | Class 1 | 17.6 | 17.3 | 19.0 |
| Training | Class 2 | 38.7 | 28.3 | 39.0 |
| Training | Class 3 | 32.5 | 39.6 | 38.3 |
| Qinghai external | Class 1 | 17.0 | 19.0 | 21.5 |
| Qinghai external | Class 2 | 27.5 | 25.0 | 43.3 |
| Qinghai external | Class 3 | 25.6 | 36.6 | 32.8 |

## Supplementary Table S5. Full prespecified adjusted Cox model.

| **Term** | **HR** | **Lower 95% CI** | **Upper 95% CI** | **P value** |
| --- | --- | --- | --- | --- |
| trajectory_classClass 2 | 1.866 | 1.246 | 2.794 | 0.002 |
| trajectory_classClass 3 | 1.732 | 1.313 | 2.285 | <0.001 |
| age_years | 1.030 | 1.024 | 1.036 | <0.001 |
| sexMale | 1.057 | 0.901 | 1.239 | 0.499 |
| stroke_typeHemorrhagic stroke | 0.892 | 0.759 | 1.048 | 0.164 |
| initial_gcs | 1.010 | 0.975 | 1.047 | 0.577 |
| bun_t1_mg_dl | 1.011 | 1.006 | 1.016 | <0.001 |
| creatinine_t1_mg_dl | 1.014 | 0.961 | 1.069 | 0.618 |
| bun_cr_ratio_t1 | 0.994 | 0.982 | 1.005 | 0.287 |

## Supplementary Table S6A. Proportional-hazards diagnostics.

| **Term** | **Chi-square** | **df** | **P value** |
| --- | --- | --- | --- |
| trajectory_class | 1.409 | 2 | 0.4943 |
| age_years | 0.007 | 1 | 0.9315 |
| sex | 0.477 | 1 | 0.4900 |
| stroke_type | 9.840 | 1 | 0.0017 |
| initial_gcs | 0.004 | 1 | 0.9494 |
| bun_t1_mg_dl | 2.453 | 1 | 0.1173 |
| creatinine_t1_mg_dl | 0.246 | 1 | 0.6199 |
| bun_cr_ratio_t1 | 3.006 | 1 | 0.0829 |
| GLOBAL | 13.710 | 9 | 0.1330 |

## Supplementary Table S6B. Multicollinearity diagnostics.

| **Term** | **Adjusted VIF/GVIF** |
| --- | --- |
| trajectory_class | 1.150 |
| age_years | 1.040 |
| sex | 1.039 |
| stroke_type | 1.019 |
| initial_gcs | 1.014 |
| bun_t1_mg_dl | 1.658 |
| creatinine_t1_mg_dl | 1.558 |
| bun_cr_ratio_t1 | 1.583 |

## Supplementary Table S6C. Functional-form diagnostics for continuous Cox covariates.

| **Variable** | **Alternative functional form** | **df difference** | **Likelihood-ratio chi-square** | **P value** |
| --- | --- | --- | --- | --- |
| age_years | Natural spline versus numeric linear term | 2 | 0.028 | 0.986 |
| initial_gcs | Unrestricted observed-support factor versus numeric linear term | 11 | 31.418 | <0.001 |
| bun_t1_mg_dl | Natural spline versus numeric linear term | 2 | 32.308 | <0.001 |
| creatinine_t1_mg_dl | Natural spline versus numeric linear term | 2 | 29.315 | <0.001 |
| bun_cr_ratio_t1 | Natural spline versus numeric linear term | 2 | 3.314 | 0.191 |

## Supplementary Table S7. Stroke-subtype-specific Cox results.

| **Stroke subtype** | **Contrast** | **HR** | **Lower 95% CI** | **Upper 95% CI** | **P value** | **Global trajectory P** |
| --- | --- | --- | --- | --- | --- | --- |
| Hemorrhagic stroke | trajectory_classClass 2 | 3.028 | 1.621 | 5.655 | <0.001 | <0.001 |
| Hemorrhagic stroke | trajectory_classClass 3 | 2.224 | 1.460 | 3.389 | <0.001 | <0.001 |
| Ischemic stroke | trajectory_classClass 2 | 1.344 | 0.794 | 2.274 | 0.271 | 0.106 |
| Ischemic stroke | trajectory_classClass 3 | 1.488 | 1.031 | 2.146 | 0.034 | 0.106 |

The formal trajectory-by-stroke-subtype interaction had P=0.383. Subtype-specific models are supportive; effect modification is judged from the interaction test rather than by comparing within-subtype P values.

## Supplementary Table S8. Alternative trajectory specifications: global Cox tests.

| **Model** | **Time form** | **Classes** | **Global trajectory P** | **Concordance** |
| --- | --- | --- | --- | --- |
| quadratic_k2 | quadratic | 2 | 0.0043 | 0.658 |
| quadratic_k3 | quadratic | 3 | 1.70e-04 | 0.660 |
| quadratic_k4 | quadratic | 4 | 9.79e-08 | 0.667 |
| quadratic_k5 | quadratic | 5 | 1.70e-05 | 0.665 |
| linear_k2 | linear | 2 | 0.0046 | 0.658 |
| linear_k3 | linear | 3 | 1.03e-07 | 0.666 |
| linear_k4 | linear | 4 | 1.52e-07 | 0.666 |

## Supplementary Table S9. Training-only ranking of eight candidate algorithms.

| **Rank** | **Model** | **Mean CV AUROC** | **SE** | **Pooled OOF PR-AUC** | **Pooled OOF Brier** | **Calibration intercept** | **Calibration slope** | **Within 1-SE set** |
| --- | --- | --- | --- | --- | --- | --- | --- | --- |
| 1 | LightGBM | 0.740 | 0.005 | 0.420 | 0.158 | 0.030 | 0.900 | Yes |
| 2 | Random Forest | 0.735 | 0.005 | 0.414 | 0.158 | -0.040 | 1.170 | No |
| 3 | KNN | 0.715 | 0.006 | 0.394 | 0.162 | 0.177 | 0.885 | No |
| 4 | XGBoost | 0.720 | 0.005 | 0.397 | 0.163 | 0.005 | 1.572 | No |
| 5 | Decision Tree | 0.669 | 0.007 | 0.365 | 0.167 | 0.007 | 0.824 | No |
| 6 | SVM | 0.694 | 0.006 | 0.383 | 0.168 | 0.009 | 1.059 | No |
| 7 | Neural Network | 0.703 | 0.007 | 0.391 | 0.183 | -0.689 | 2.200 | No |
| 8 | Naive Bayes | 0.719 | 0.006 | 0.383 | 0.206 | -0.067 | 0.255 | No |

## Supplementary Table S10. Performance at the frozen training-derived thresholds.

| **Cohort** | **Feature set** | **Locked threshold** | **Sensitivity** | **Specificity** | **PPV** | **NPV** | **Accuracy** | **F1** |
| --- | --- | --- | --- | --- | --- | --- | --- | --- |
| Internal test | M2 | 0.221 | 0.695 | 0.648 | 0.376 | 0.874 | 0.659 | 0.488 |
| Qinghai external | M0 | 0.222 | 0.224 | 0.869 | 0.367 | 0.768 | 0.705 | 0.278 |
| Qinghai external | M1 | 0.193 | 0.296 | 0.817 | 0.354 | 0.774 | 0.685 | 0.322 |
| Qinghai external | M2 | 0.221 | 0.255 | 0.879 | 0.417 | 0.777 | 0.721 | 0.316 |

## Supplementary Table S11. Probability performance of all eight algorithms.

| **Cohort** | **Model** | **AUROC** | **Lower 95% CI** | **Upper 95% CI** | **PR-AUC** | **Brier** |
| --- | --- | --- | --- | --- | --- | --- |
| Internal test | LightGBM | 0.734 | 0.695 | 0.773 | 0.455 | 0.157 |
| Internal test | Random Forest | 0.740 | 0.702 | 0.779 | 0.476 | 0.156 |
| Internal test | KNN | 0.715 | 0.676 | 0.755 | 0.422 | 0.161 |
| Internal test | XGBoost | 0.678 | 0.636 | 0.721 | 0.425 | 0.178 |
| Internal test | Decision Tree | 0.638 | 0.591 | 0.684 | 0.357 | 0.173 |
| Internal test | SVM | 0.702 | 0.661 | 0.743 | 0.413 | 0.166 |
| Internal test | Neural Network | 0.701 | 0.660 | 0.742 | 0.427 | 0.182 |
| Internal test | Naive Bayes | 0.715 | 0.674 | 0.755 | 0.416 | 0.198 |
| Qinghai external | LightGBM | 0.624 | 0.559 | 0.689 | 0.341 | 0.199 |
| Qinghai external | Random Forest | 0.634 | 0.570 | 0.698 | 0.384 | 0.185 |
| Qinghai external | KNN | 0.547 | 0.480 | 0.614 | 0.328 | 0.205 |
| Qinghai external | XGBoost | 0.630 | 0.566 | 0.693 | 0.353 | 0.188 |
| Qinghai external | Decision Tree | 0.426 | 0.371 | 0.482 | 0.211 | 0.211 |
| Qinghai external | SVM | 0.546 | 0.476 | 0.616 | 0.319 | 0.189 |
| Qinghai external | Neural Network | 0.576 | 0.514 | 0.638 | 0.313 | 0.200 |
| Qinghai external | Naive Bayes | 0.614 | 0.550 | 0.679 | 0.394 | 0.212 |

## Supplementary Table S12. Complete global TreeSHAP importance in the internal test set.

| **Feature** | **Mean absolute SHAP** |
| --- | --- |
| age_years | 0.4872 |
| initial_glucose_mg_dl | 0.2963 |
| delirium | 0.2071 |
| initial_total_calcium_mg_dl | 0.1888 |
| urine_output_first_24h_ml | 0.1493 |
| initial_wbc_10e9_l | 0.1411 |
| bun_t1_mg_dl | 0.1265 |
| initial_rbc_10e12_l | 0.1200 |
| initial_heart_rate_bpm | 0.1055 |
| initial_respiratory_rate_breaths_min | 0.1022 |
| initial_potassium_mmol_l | 0.0924 |
| creatinine_t1_mg_dl | 0.0810 |
| initial_anion_gap_mmol_l | 0.0770 |
| bun_cr_ratio_t1 | 0.0530 |
| congestive_heart_failure | 0.0436 |
| acute_kidney_injury | 0.0273 |
| trajectory_class | 0.0232 |
| severe_liver_disease | 0.0196 |
| stroke_type | 0.0049 |
| crrt | 0.0048 |
| chronic_renal_disease | 0.0012 |

## Supplementary Table S13. Prespecified delirium-handling sensitivity analyses.

| **Scenario** | **Feature set** | **AUROC** | **Lower 95% CI** | **Upper 95% CI** | **PR-AUC** | **Brier** | **Calibration intercept** | **Calibration slope** |
| --- | --- | --- | --- | --- | --- | --- | --- | --- |
| delirium_mode_impute | M1 | 0.723 | 0.683 | 0.762 | 0.468 | 0.158 | 0.012 | 1.065 |
| delirium_mode_impute | M2 | 0.720 | 0.680 | 0.759 | 0.455 | 0.159 | 0.019 | 0.852 |
| remove_delirium | M1 | 0.718 | 0.679 | 0.758 | 0.455 | 0.159 | 0.012 | 1.071 |
| remove_delirium | M2 | 0.715 | 0.675 | 0.754 | 0.437 | 0.161 | 0.030 | 0.847 |

## Supplementary Table S14. Diagnosis codes used to define acute stroke subtypes.

| **Study subtype** | **Code system** | **Code family** | **Exact included codes (decimal points omitted)** |
| --- | --- | --- | --- |
| Nontraumatic subarachnoid hemorrhage (SAH) | ICD-10-CM / ICD-9-CM | I60* / 430 | I6000, I6001, I6002, I6010, I6011, I6012, I602, I6030, I6031, I6032, I604, I6050, I6051, I6052, I606, I607, I608, I609, 430 |
| Nontraumatic intracerebral hemorrhage (ICH) | ICD-10-CM / ICD-9-CM | I61* / 431 | I61, I610, I611, I612, I613, I614, I615, I616, I618, I619, 431 |
| Cerebral infarction / ischemic stroke | ICD-10-CM / ICD-9-CM | I63* / 433x1 / 434x1 | I63, I630, I631, I632, I633, I634, I635, I636, I638, I639, I6300, I6301, I6302, I6303, I6309, I6310, I6311, I6312, I6313, I6319, I6320, I6321, I6322, I6323, I6329, I6330, I6331, I6332, I6333, I6334, I6339, I6340, I6341, I6342, I6343, I6344, I6349, I6350, I6351, I6352, I6353, I6354, I6359, I6381, I6389, I63011, I63012, I63013, I63019, I63031, I63032, I63033, I63039, I63111, I63112, I63113, I63119, I63131, I63132, I63133, I63139, I63211, I63212, I63213, I63219, I63231, I63232, I63233, I63239, I63311, I63312, I63313, I63319, I63321, I63322, I63323, I63329, I63331, I63332, I63333, I63339, I63341, I63342, I63343, I63349, I63411, I63412, I63413, I63419, I63421, I63422, I63423, I63429, I63431, I63432, I63433, I63439, I63441, I63442, I63443, I63449, I63511, I63512, I63513, I63519, I63521, I63522, I63523, I63529, I63531, I63532, I63533, I63539, I63541, I63542, I63543, I63549, 43301, 43311, 43321, 43331, 43381, 43391, 43401, 43411, 43491 |

Note: MIMIC-IV diagnosis codes are stored without decimal points. The exact list above reproduces the code set supplied for cohort extraction. ICH indicates intracerebral hemorrhage; SAH, subarachnoid hemorrhage.

## Supplementary Table S15. Pre-imputation missingness in standardized analysis variables.

| **Variable** | **MIMIC-IV n** | **MIMIC missing, n (%)** | **Qinghai n** | **Qinghai missing, n (%)** |
| --- | --- | --- | --- | --- |
| Age, years | 2805 | 0 (0.0%) | 387 | 0 (0.0%) |
| Male sex | 2805 | 0 (0.0%) | 387 | 0 (0.0%) |
| Hemorrhagic stroke | 2805 | 0 (0.0%) | 387 | 0 (0.0%) |
| Initial GCS | 2805 | 7 (0.2%) | 387 | 0 (0.0%) |
| Initial heart rate, beats/min | 2805 | 6 (0.2%) | 387 | 0 (0.0%) |
| Initial systolic blood pressure, mmHg | 2805 | 6 (0.2%) | 387 | 0 (0.0%) |
| Initial diastolic blood pressure, mmHg | 2805 | 6 (0.2%) | 387 | 0 (0.0%) |
| Initial mean arterial pressure, mmHg | 2805 | 6 (0.2%) | 387 | 0 (0.0%) |
| Initial respiratory rate, breaths/min | 2805 | 12 (0.4%) | 387 | 0 (0.0%) |
| Initial temperature, C | 2805 | 79 (2.8%) | 387 | 0 (0.0%) |
| Initial chloride, mmol/L | 2805 | 1 (0.0%) | 387 | 0 (0.0%) |
| Initial anion gap, mmol/L | 2805 | 4 (0.1%) | 387 | 18 (4.7%) |
| Initial bicarbonate, mmol/L | 2805 | 1 (0.0%) | 387 | 14 (3.6%) |
| Initial total calcium, mg/dL | 2805 | 120 (4.3%) | 387 | 0 (0.0%) |
| Initial potassium, mmol/L | 2805 | 1 (0.0%) | 387 | 0 (0.0%) |
| Initial sodium, mmol/L | 2805 | 1 (0.0%) | 387 | 0 (0.0%) |
| Initial glucose, mg/dL | 2805 | 5 (0.2%) | 387 | 0 (0.0%) |
| Initial white blood cell count, 10^9/L | 2805 | 12 (0.4%) | 387 | 0 (0.0%) |
| Initial red blood cell count, 10^12/L | 2805 | 13 (0.5%) | 387 | 0 (0.0%) |
| Initial platelet count, 10^9/L | 2805 | 14 (0.5%) | 387 | 0 (0.0%) |
| Urine output in first 24 h, mL | 2805 | 85 (3.0%) | 387 | 5 (1.3%) |
| Congestive heart failure | 2805 | 0 (0.0%) | 387 | 0 (0.0%) |
| Chronic pulmonary disease | 2805 | 0 (0.0%) | 387 | 0 (0.0%) |
| Diabetes | 2805 | 0 (0.0%) | 387 | 0 (0.0%) |
| Chronic renal disease | 2805 | 0 (0.0%) | 387 | 0 (0.0%) |
| Severe liver disease | 2805 | 0 (0.0%) | 387 | 0 (0.0%) |
| Delirium | 2805 | 451 (16.1%) | 387 | 1 (0.3%) |
| Acute kidney injury | 2805 | 0 (0.0%) | 387 | 0 (0.0%) |
| CRRT | 2805 | 0 (0.0%) | 387 | 0 (0.0%) |
| T1 BUN, mg/dL | 2805 | 0 (0.0%) | 387 | 0 (0.0%) |
| T1 creatinine, mg/dL | 2805 | 0 (0.0%) | 387 | 0 (0.0%) |
| T1 BUN/Cr ratio | 2805 | 0 (0.0%) | 387 | 0 (0.0%) |
| T2 BUN/Cr ratio | 2805 | 0 (0.0%) | 387 | 0 (0.0%) |
| T3 BUN/Cr ratio | 2805 | 0 (0.0%) | 387 | 0 (0.0%) |
| SAPS II score | 2805 | 0 (0.0%) | 387 | 0 (0.0%) |
| SOFA score | 2805 | 0 (0.0%) | 387 | 0 (0.0%) |

Note: Missingness is reported before machine-learning preprocessing. All standardized analysis variables shown in the table are retained, including variables with zero missing values. Most variables had less than 5% missingness; delirium was missing in 451/2,805 MIMIC-IV patients (16.1%).

## Supplementary Table S16. Post hoc descriptive comparison of the strict 72-hour landmark cohort and mutually exclusive excluded groups.

| **Characteristic** | **Landmark included (n=2,805)** | **Observable at 72 h, incomplete T1-T3 (n=864)** | **SMD** | **Left before 72 h, ICU-death flag (n=387)** | **SMD** | **Left before 72 h, no ICU-death flag (n=2,934)** | **SMD** |
| --- | --- | --- | --- | --- | --- | --- | --- |
| Age, years | 67.7 [56.0, 78.5] | 69.0 [58.2, 79.9] | 0.092 | 73.8 [62.6, 84.4] | 0.380 | 72.0 [60.6, 82.5] | 0.256 |
| Male sex | 1516 (54.0%) | 447 (51.7%) | 0.046 | 194 (50.1%) | 0.078 | 1449 (49.4%) | 0.093 |
| Hemorrhagic stroke | 1208 (43.1%) | 340 (39.4%) | 0.076 | 255 (65.9%) | 0.471 | 1121 (38.2%) | 0.099 |
| Initial GCS | 15.0 [14.0, 15.0] | 15.0 [14.0, 15.0] | 0.002 | 15.0 [15.0, 15.0] | 0.075 | 15.0 [14.0, 15.0] | 0.044 |
| SAPS II score | 34.0 [26.0, 43.0] | 33.0 [25.0, 42.0] | 0.085 | 43.0 [35.0, 56.0] | 0.701 | 31.0 [24.0, 38.0] | 0.318 |
| SOFA score | 4.0 [2.0, 6.0] | 3.0 [1.0, 5.0] | 0.195 | 5.0 [3.0, 8.0] | 0.344 | 2.0 [1.0, 4.0] | 0.509 |
| Glucose, mg/dL | 132.0 [109.0, 166.0] | 129.0 [105.0, 163.0] | 0.046 | 151.0 [122.5, 204.5] | 0.294 | 118.0 [100.0, 147.0] | 0.225 |
| WBC, 10^9/L | 11.1 [8.4, 14.5] | 10.1 [7.8, 13.1] | 0.130 | 12.8 [9.5, 17.4] | 0.204 | 9.2 [7.2, 11.9] | 0.216 |
| Urine output first 24 h, mL | 1575.0 [989.5, 2369.2] | 1433.5 [900.0, 2200.0] | 0.098 | 1430.0 [693.8, 2533.8] | 0.088 | 1400.0 [873.8, 2050.0] | 0.222 |
| T1 BUN/Cr ratio | 17.62 [13.75, 22.73] | 16.67 [13.33, 21.51] | 0.125 | 18.33 [15.00, 23.83] | 0.088 | 18.33 [14.29, 23.00] | 0.020 |
| Delirium | 1497 (63.6%) | 377 (49.4%) | 0.289 | 63 (61.2%) | 0.050 | 467 (21.5%) | 0.940 |
| Acute kidney injury | 2467 (88.0%) | 739 (85.5%) | 0.071 | 254 (65.6%) | 0.548 | 1419 (48.4%) | 0.939 |
| Recorded death by day 28 | 657 (23.4%) | 203 (23.5%) | 0.002 | 387 (100.0%) | 2.557 | 536 (18.3%) | 0.127 |

Note: Continuous variables are median [IQR]; categorical variables are n (%) using the observed denominator. Absolute standardized mean differences compare each excluded group with the strict landmark cohort. This post hoc analysis characterizes the direction and magnitude of selection associated with 72-hour observability and complete T1-T3 measurements; it does not remove selection bias. Absence of an ICU-death flag is not evidence of clinical stability. The two records with missing ICU outtime are reported separately in the flow diagram and omitted from this table.

# Supplementary Figures

## Supplementary Figure S1.

Supplementary Figure S1. Repeated-cross-validation AUROC estimates for the eight candidate algorithms in the training cohort. This forest plot complements, rather than duplicates, the patient-level out-of-fold ROC curves in main Figure 6A.

## Supplementary Figure S2.

Supplementary Figure S2. Pooled training out-of-fold precision-recall curves for M0, M1, and M2. Precision-recall curves are not displayed in the main figures; the horizontal reference indicates the 23.4% training event prevalence.

## Supplementary Figure S3.

Supplementary Figure S3. Internal-test ROC comparison of the primary M2 model with two prespecified alternative delirium-handling strategies using fixed LightGBM hyperparameters.

## Supplementary Figure S4.

Supplementary Figure S4. Distribution of trajectory-class TreeSHAP contributions in the internal test set.

## Supplementary Figure S5.

Supplementary Figure S5. Native LightGBM TreeSHAP dependence plots for five leading continuous predictors in the internal test set.

## Supplementary Figure S6.

Supplementary Figure S6. Native LightGBM TreeSHAP force plots for the representative higher-risk death and lower-risk survival cases shown as waterfall plots in main Figure 8.
